# Supplementary material for: Mapping of class II HLA restriction of SepSecS-specific CD4 T-cell epitopes in autoimmune hepatitis
Source: JHEP Rep. 2026 Aug 11;8(9):101952. doi: 10.1016/j.jhepr.2026.101952 (PMC13482648; doi:10.1016/j.jhepr.2026.101952)
Supplement: Supplementary file 1 [file mmc1.pdf]

Mapping of class II HLA restriction of SepSecS-specific CD4 T cell epitopes in autoimmune hepatitis

Thomas Guinebretière, Alexandra Garcia, Chuang Dong, Laura Bernier, Virginie Huchet, Pierre-Jean Gavlovsky, Laurine Gil, Sakina Ado, Léa David, Caroline Chevalier, Jean-Paul Judor, Béatrice Clémenceau, Marion Khaldi, Edouard Bardou-Jacquet, Laure Elkrief, Adrien Lannes, Christine Silvain, Matthieu Schnee, Florence Tanne, Sara Lemoinne, Eleonora De Martin, Aurélie Plessier, Fabienne Vavasseur, David-Axel Laplaud, William W. Kwok, Sophie Brouard, Jean-François Mosnier, Jérôme Gournay, Pierre Milpied, Sophie Conchon, Amédée Renand

Fig. S1..2
Table S1..3
Table S2..4
Table S3..separate excel file
Table S4..4
Table S5..separate excel file
Table S6..5
Table S7..separate excel file
Table S8..6
Supplementary references..7

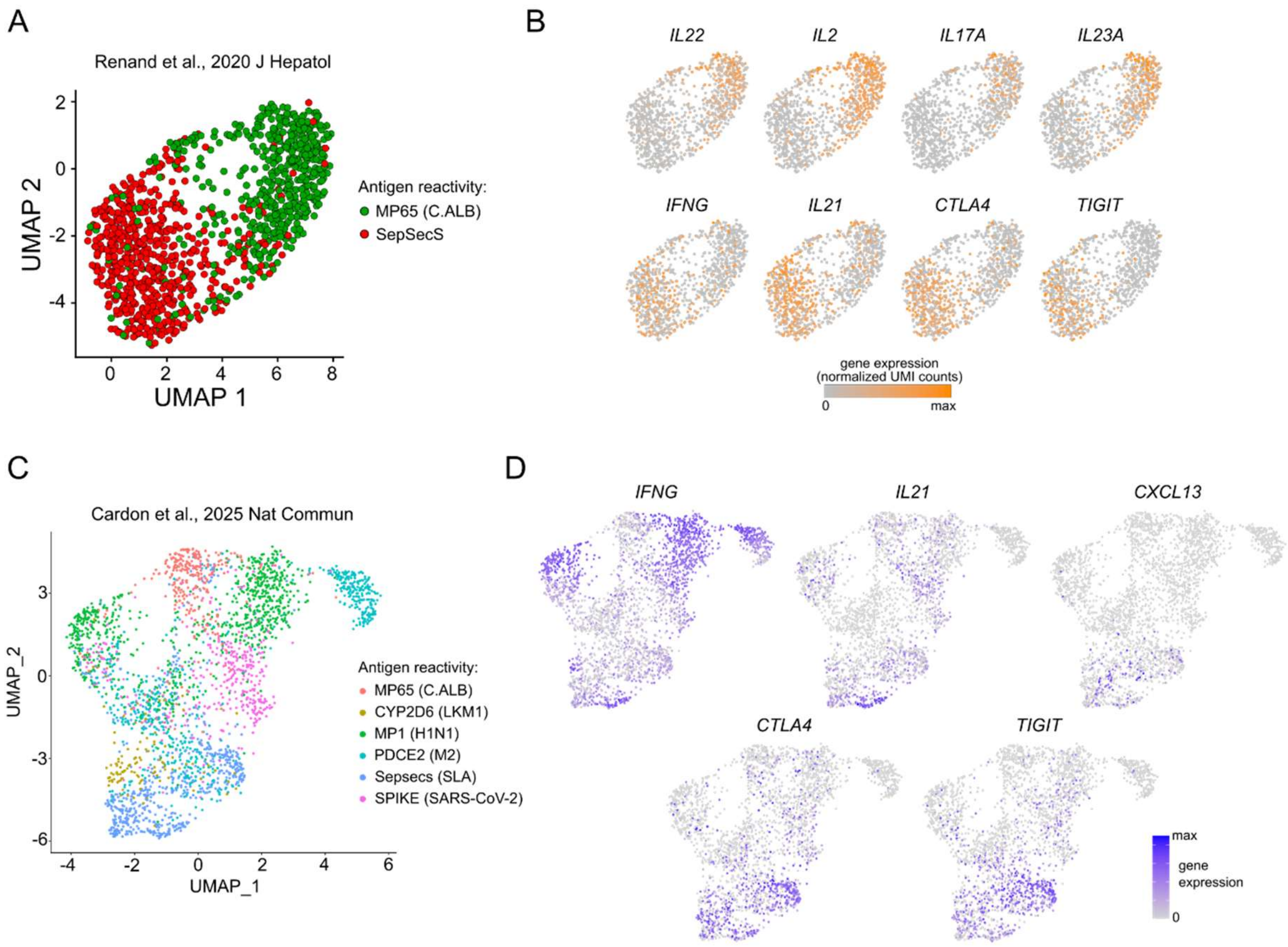

Fig. S1. Molecular profile of antigen-specific CD4 T cells from Renand et al., 2020 J Hepatol and Cardon et al., 2025 Nat Commun. (A) UMAP representation of single-cell transcriptomes of antigen-specific CD4 T cells from Renand et al., 2020 J Hepatol, colored by antigen reactivity (MP65-specific CD4 T cells in green, SepSecS-specific CD4 T cells in red). (B) UMAP representation used in A colored by expression of indicated genes. (C) UMAP representation of single-cell transcriptomes of antigen-specific CD4 T cells from Cardon et al., 2025 Nat Commun, colored by antigen reactivity as indicated. (D) UMAP representation used in C colored by expression of indicated genes.

Table S1. Amino acid sequence of SepSecS peptides.

peptide sequence (red=false, blue=substitution)	peptide number (p#)	position on AAD33963 (used in the manuscript)	position on Q9HD40 (curated sequence)	pool number	reference if published
MSTSYGCFWRRFIHGIGRSG	p1	1-20	/	1	
WRRFIHGIGRSGDISAVQPK	p2	9-28	90-107	1	
GRSGDISAVQPKAAGSSLLN	p3	17-36	96-115	1	
VQPKAAGSSLLNKITNSLVL	p4	25-44	104-123	1	
SLLNKITNSLVLDIIKLAGV	p5	33-52	112-131	1	
SLVLDIIKLAGVHTVANCFV	p6	41-60	120-139	2	[1]
LAGVHTVANCFVVPMATGMS	p7	49-68	128-147	2	
NCFVVPMATGMSLTLCFLTL	p8	57-76	136-155	2	[1]
TGMSLTLCFLTLRHKRPKAK	p9	65-84	144-163	2	[1]
FLTLRHKRPKAKYIIWPRID	p10	73-92	152-171	2	[2,3]
PKAKYIIWPRIDQKSCFKSM	p11	81-100	160-179	3	[2,3]
PRIDQKSCFKSMITAGFEPV	p12	89-108	168-187	3	[2]
FKSMITAGFEPVVIENVLEG	p13	97-116	176-195	3	[2]
FEPVVIENVLEGDELRTDLK	p14	105-124	184-203	3	[2]
VLEGDELRTDLKAVEAKVQE	p15	113-144	192-211	3	
TDLKAVEAKVQELGPDCILC	p16	121-140	200-219	4	
KVQELGPDCILCIHSTTSCF	p17	129-148	208-227	4	
CILCIHSTTSCFAPRVPDRL	p18	137-156	216-235	4	
TSCFAPRVPDRLEELAVICA	p19	145-164	224-243	4	
PDRLEELAVICANYDIPHIV	p20	153-173	232-251	4	
VICANYDIPHIVNNAYGVQS	p21	161-180	240-259	5	
PHIVNNAYGVQSSKCMHLIQ	p22	169-188	248-267	5	
GVQSSKCMHLIQQGARVGRI	p23	177-196	256-275	5	[2–5]
HLIQQGARVGRIDAFVQSLD	p24	185-204	264-283	5	[2–5]
VGRIDAFVQSLDKNFMVPVG	p25	193-212	272-291	5	
QSLDKNFMVPVGGAIIAGFN	p26	201-220	280-299	6	
VPVGGAIIAGFNDSFIQEIS	p27	209-228	288-307	6	
AGFNDSFIQEISKMYPGRAS	p28	217-236	296-315	6	
QEISKMYPGRASASPSLDVL	p29	225-244	304-323	6	
GRASASPSLDVLITLLSLGS	p30	233-252	312-331	6	
LDVLITLLSLGSNGYKKLLK	p31	241-260	320-339	7	
SLGSNGYKKLLKERKEMFSY	p32	249-268	328-347	7	
KLLKERKEMFSYLSNQIKKL	p33	257-276	336-355	7	[4]
MFSYLSNQIKKLSEAYNERL	p34	265-284	344-363	7	
IKKLSEAYNERLLHTPHNPI	p35	273-292	352-371	7	
NERLLHTPHNPISLAMTLKT	p36	281-300	360-379	8	
HNPISLAMTLKTLDEHRDKA	p37	289-308	368-387	8	
TLKTLDEHRDKAVTQLGSML	p38	297-316	376-395	8	
RDKAVTQLGSMLFTKQVSGA	p39	305-324	384-403	8	[1]
GSMLFTKQVSGARVVPLGSM	p40	313-332	392-411	8	
VSGARVVPLGSMQTVSGYTF	p41	321-340	400-419	9	[1]
LGSMQTVSGYTFRGFMSHTN	p42	329-348	408-427	9	
GYTFRGFMSHTNNYPCAYLN	p43	337-356	416-435	9	
SHTNNYPCAYLNAASAIGMK	p44	345-364	424-443	9	
AYLNAASAIGMKMQDVDLFI	p45	353-372	432-451	9	
IGMKMQDVDLFINRLDRCLK	p46	361-380	440-459	10	
DLFINRLDRCLKAVRKERSK	p47	369-388	448-467	10	[4]
RCLKAVRKERSKESDDNYDK	p48	377-396	456-475	10	
ERSKESDDNYDKTEDVDIEE	p49	385-404	464-483	10	
NYDKTEDVDIEEMALKLDNV	p50	393-412	472-491	10	
DIEEMALKLDNVLLDTYQDA	p51	401-420	480-499	11	
LDNVLLDTYQDASS	p52	409-422	488-501	11	
EEMALKLDNVLLDTYQDASS	p53	403-422	482-501	11	

Table S2. List of antibodies.

antibody name	clone	antigen	brand	reference	dilution
Alexa Fluor 488 anti-human CD4	OKT4	CD4	BioLegend	317420	20
PE anti-human CD154	5C8	CD154	Miltenyi	130-113-607	20
PerCP/Cyanine5.5 anti-human CD279 (PD-1)	EH12.2H7	CD279	BioLegend	329914	20
APC/Cyanine7 anti-human CD45RA	HI100	CD45RA	BioLegend	304127	20
Brilliant Violet 510 anti-human CD3	OKT3	CD3	BioLegend	317332	20

Table S3. List of TCR clonotypes from scRNA-seq data.

See Table S3.xlsx.

Table S4. Class II HLA genotypes of Epstein-Barr Virus B lymphoblastoid cell lines (BLCL).

BLCL ID	HLA-DRB1	HLADQB1	HLADPA1	HLADPB1
BLCL FEB*DR3	03:01 / 03:01	02:01 / 02:01	NA	04:01 / 04:01
BLCL 1064	03:01 / 07:01	02:01 / 02:02	01:03 / 02:01	01:01 / 04:01

NA: no data available.

Table S5. Epitope binding predictions using NetMHCIIpan 4.1 BA tool.

See Table S5.xlsx.

Table S6. GLIPH2 clustering of TCRs from SepSecS$_{187-197}$-loaded HLA-DRB1*03:01 tetramer-specific CD4 T cells and clones recognizing SepSecS$_{177-204}$ epitope *in vitro.*

Group	TCR cell line	Epitope	index	Pattern	CDR3β	Vβ	Jβ	CDR3α	Patient ID
			$1_{2,3,4,5}$	(Q)GVYG	CASSESS**QGVYG**YTF	TRBV6-1	TRBJ1-2	NA	01-004
			$1_{4,5}$	GVYG	CATSSQTG**GVYG**YTF	TRBV15	TRBJ1-2	CAVDRGSGYSTLTF	01-004
			$1_{4,5}$	GVYG	CATSSQTG**GVYG**YTF	TRBV15	TRBJ1-2	CAVGSGGYNKLIF	01-004
			$1_{4,5}$	GVYG	CATSSQTG**GVYG**YTF	TRBV15	TRBJ1-2	NA	01-004
I			$1_{2,3,4,5}$	(Q)GVYG	CASSSTR**QGVYG**YTF	TRBV7-9	TRBJ1-2	CAFTNFGNEKLTF	01-004
			$1_{2,3,4,5}$	(Q)GVYG	CASSSTR**QGVYG**YTF	TRBV7-9	TRBJ1-2	NA	01-128
			$1_{2,3,4,5}$	(Q)GVYG	CASSSTR**QGVYG**YTF	TRBV7-9	TRBJ1-2	CAVGDAGYSTLTF	01-004
	clone 6 2020	SepSecS$_{177-204}$ (p23/24)	$1_{2,3,4,5}$	(Q)GVYG	CASSFPG**QGVYG**YTF	TRBV7-2	TRBJ1-2	CAVGSGGYNKLIF	01-018
			3	QGV	CATSSPG**QGV**QPQHF	TRBV15	TRBJ1-5	CAVGPGGYNKLIF	01-018
II	clone 7 2020	SepSecS$_{177-204}$ (p23/24)	8	single	CASSSFDRGNYGYTF	TRBV7-3	TRBJ1-2	CAVTPSGGYQKVTF	01-018
			8	single	CASSSFDRGNYGYTF	TRBV7-3	TRBJ1-2	CAGGPNKLIF	01-018
			41	single	CASSRRLVSSYNEQFF	TRBV5-4	TRBJ2-1	CAASMVDTGRRALTF	01-128
III			41	single	CASSRRLVSSYNEQFF	TRBV5-4	TRBJ2-1	CAVSPNFGNEKLTF	01-128
			41	single	CASSRRLVSSYNEQFF	TRBV5-4	TRBJ2-1	NA	01-128
IV			52	single	CSARYPLRLGNTIYF	TRBV20-1	TRBJ1-3	CAVDGANDYKLSF	01-046
			52	single	CSARYPLRLGNTIYF	TRBV20-1	TRBJ1-3	NA	01-004

index: X_Y where X is the principal index (red pattern) and Y is the additional index(es) (blue pattern); single: exact same CDR3β sequence; NA: no data available.

Table S7. Output data from GLIPH2 TCR clustering analysis shown in Fig. 3A.

See Table S7.xlsx.

Table S8. Clinical characteristics and SepSecS reactivity of patients used in Fig. 4.

Patient ID	*DP*2.1*	Age	Sex	AST (UI/L)	ALT (UI/L)	IgG (g/L)	Treatment	Nb CD154+ PD-1+ mCD4 per 10^6 mCD4		
								$SepSecS_{total}$	$SepSecS_{73-100}$	none
01-051	no	36	F	47	52	17,3	Azathioprin	1166	48	12
05-004	no	45	F	21	17	11,8	Steroids / MMF	223	0	0
07-009	no	54	F	58	58	45	Azathioprin	248	18	6
08-002	no	45	F	20	20	ND	Azathioprin	366	84	0
01-354	no	59	F	115	167	18	No treatment	97	11	0
05-001	no	58	F	38	32	16,8	MMF	163	47	1
01-046	yes	65	F	21	13	10,5	Azathioprin	179	27	10
01-165	yes	34	F	53	104	16,6	No treatment	1478	586	34
01-018	yes	38	F	60	64	20,2	Azathioprin	1200	691	7
01-004	yes	39	F	21	16	13,4	Steroids / Azathioprin	2348	349	4
01-128	yes	33	F	21	18	13,1	Azathioprin	145	98	0

*DP*2.1*: *HLA-DPA1*02:01~HLA-DPB1*01:01*; AST: aspartate transaminase; ALT: alanine transaminase; IgG: type G immunoglobulin; Nb: number; mCD4: CD45RA- CD4+ CD3+ T cells; F: female; MMF: mycophenolate mofetil; ND: not determined.

Supplementary references

Author names in bold designate shared co-first authorship

[1] **Zhao Y**, **Zhang Y**, Liu Y, et al. Identification of T cell epitopes on soluble liver antigen in Chinese patients with auto-immune hepatitis. Liver Int 2011;31:721–9.

[2] Kramer M, Mele F, Jovic S, et al. Clonal analysis of SepSecS-specific B and T cells in autoimmune hepatitis. J Clin Invest 2025;135:e183776.

[3] Renand A, Cervera-Marzal I, Gil L, et al. Integrative molecular profiling of autoreactive CD4 T cells in autoimmune hepatitis. J Hepatol 2020;73:1379–90.

[4] **Mix H**, **Weiler–Normann C**, Thimme R, et al. Identification of CD4 T-Cell Epitopes in Soluble Liver Antigen/Liver Pancreas Autoantigen in Autoimmune Hepatitis. Gastroenterology 2008;135:2107–18.

[5] **Cardon A**, **Guinebretière T**, Dong C, et al. Single cell profiling of circulating autoreactive CD4 T cells from patients with autoimmune liver diseases suggests tissue imprinting. Nat Commun 2025;16:1161.
