# Supplementary material for: Mapping of class II HLA restriction of SepSecS-specific CD4 T-cell epitopes in autoimmune hepatitis
Source: JHEP Rep. 2026 Aug 11;8(9):101952. doi: 10.1016/j.jhepr.2026.101952 (PMC13482648; doi:10.1016/j.jhepr.2026.101952)
Supplement: Supplementary file 3 [file mmc7.pdf]

Research article

JHEP Reports

Mapping of class II HLA restriction of SepSecS-specific CD4 T-cell epitopes in autoimmune hepatitis

Authors

Thomas Guinebretière, Alexandra Garcia, Chuang Dong, …, Pierre Milpied, Sophie Conchon, Amédée Renand

Correspondence

amedee.renand@univ-nantes.fr (A. Renand).

Graphical abstract

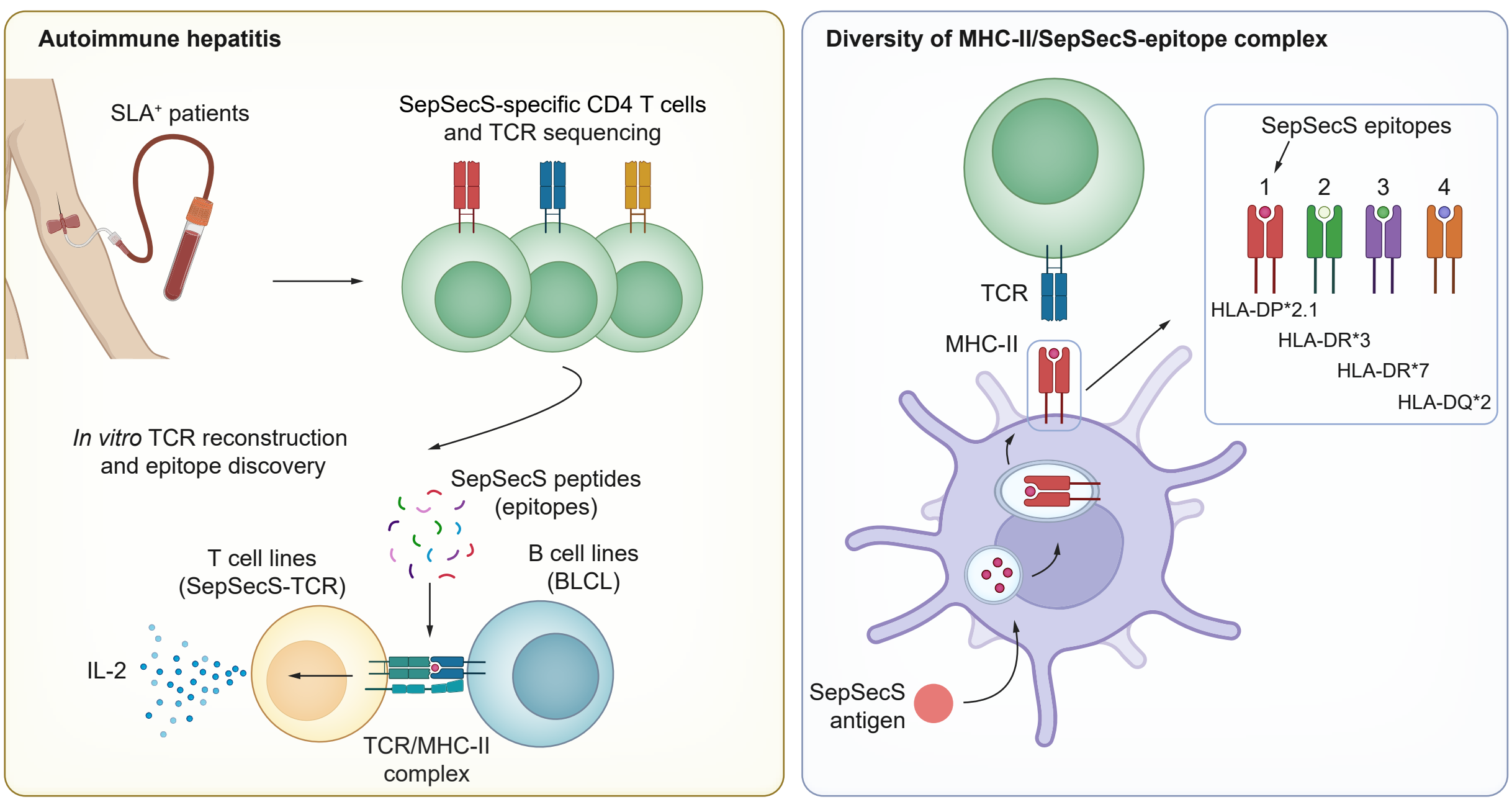

Highlights:

- Autoreactive TCRs recognize SepSecS epitopes on HLA-DR and non-HLA-DR molecules.
- Shared CDR3 motif suggests common self-epitope recognition by SepSecS-specific TCRs.
- $SepSecS_{152-179}$ reactivity is higher in *HLA-DPA1*02:01* ∼*HLA-DPB1*01:01* AIH patients.

Impact and implications:

In AIH, SepSecS is a key autoantigen targeted by the adaptive immune system, but only few T-cell epitopes are defined and associated with a precise HLA restriction. By reconstructing TCRs from the most expanded SepSecS-specific CD4 T cells of AIH patients, we identified new T-cell epitopes and their specific class II HLA restriction, including peptides presented by non-HLA-DR molecules. From a clinical perspective, these findings could be used to develop tools to track the frequency of autoreactive T cells in patients. This work could also pave the way for self-antigen-specific immunotherapies, such as peptide-based desensitization or the development of HLA-multimer technologies to selectively deplete or reprogram pathogenic T cells without inducing systemic immunosuppression.

https://doi.org/10.1016/j.jhepr.2026.101952
© 2026 The Author(s). Published by Elsevier B.V. on behalf of European Association for the Study of the Liver (EASL). This is an open access article under the CC BY license (http://creativecommons.org/licenses/by/4.0/). JHEP Reports, 2026, 8, 1–14

JHEP|Reports

Mapping of class II HLA restriction of SepSecS-specific CD4 T-cell epitopes in autoimmune hepatitis

Thomas Guinebretière[1], Alexandra Garcia[1], Chuang Dong[2], Laura Bernier[1], Virginie Huchet[1], Pierre-Jean Gavlovsky[1], Laurine Gil[2], Sakina Ado[2], Léa David[2], Caroline Chevalier[3], Jean-Paul Judor[1], Béatrice Clémenceau[4], Marion Khaldi[5,6], Edouard Bardou-Jacquet[7], Laure Elkrief[8], Adrien Lannes[9], Christine Silvain[10], Matthieu Schnee[11], Florence Tanne[12], Sara Lemoinne[13,14,15], Eleonora De Martin[16], Aurélie Plessier[17], Fabienne Vavasseur[3], David-Axel Laplaud[1,18], William W. Kwok[19], Sophie Brouard[1], Jean-François Mosnier[20], Jérôme Gournay[1,5,6], Pierre Milpied[2], Sophie Conchon[1], Amédée Renand[1,*]

JHEP Reports **2026**. vol. 8 | 1–14

Check for updates

Background & Aims: Autoreactive CD4 T cells, which recognize liver self-antigens such as SepSecS, are potentially main drivers of the chronic inflammatory response during autoimmune hepatitis (AIH). Previous studies have revealed SepSecS epitopes often associated with HLA-DRB1*03 or HLA-DRB1*04 restriction, two alleles enriched in AIH population. However, human leukocyte antigen (HLA) restriction of numerous SepSecS epitopes remains incomplete, and whether they could be presented by non-HLA-DR molecules remains unclear. Here, we investigated epitope recognition and HLA restriction of SepSecS-specific CD4 T cells from AIH patients.

Methods: We cloned 17 T-cell receptor αβ (TCRαβ) from the most expanded SepSecS-specific CD4 T cells of five AIH patients from our previous studies into a murine hybridoma T-cell line. The epitope reactivity of each TCR cell line was identified via IL-2 secretion following culture with 20-mer overlapping peptides of the SepSecS protein and immortalized B-cell lines. HLA restriction was defined using HLA blocking antibodies. We analyzed 484 SepSecS-specific TCRs from nine AIH patients using GLIPH2 algorithm. CD154 activation-induced marker assay was used to evaluate the SepSecS epitope reactivity in AIH patients (n = 11).

Results: Autoreactive TCRs recognized six SepSecS amino acid regions *in vitro*, with four distinct class II HLA restrictions, including non-HLA-DR restrictions. The GLIPH2 algorithm clustered SepSecS-specific TCRs into six groups. Notably, 6% of the total SepSecS-specific TCR clonotypes shared common CDR3β motifs linked to the recognition of HLA-DPA1*02:01/HLA-DPB1*01:01-restricted $\text{SepSecS}_{152\text{-}179}$ epitope. *Ex vivo* peptide stimulation assay revealed that $\text{SepSecS}_{152\text{-}179}$ epitope represents 15% to 67% of total SepSecS CD4 T-cell reactivity in *HLA-DPA1*02:01∼HLA-DPB1*01:01* AIH patients (n = 5).

Conclusions: We highlighted that SepSecS epitopes could be presented by HLA-DR and non-HLA-DR molecules to autoreactive CD4 TCRs with potentially conserved CDR3 motifs for common epitope recognition.

© 2026 The Author(s). Published by Elsevier B.V. on behalf of European Association for the Study of the Liver (EASL). This is an open access article under the CC BY license (http://creativecommons.org/licenses/by/4.0/).

Introduction

Autoimmune hepatitis (AIH) is a chronic inflammatory liver disease affecting all age groups and ethnicities, with a predominant occurrence in women.[1] The increased incidence and prevalence in the past years coupled with frequent relapses after immunosuppressive drug withdrawal highlight the critical need to elucidate AIH pathogenesis.[1–3] Classically, specific autoantibodies help to distinguish subtypes of AIH. Antinuclear and anti-smooth muscle antibodies are associated with the most common variant type 1 AIH (AIH-1), whereas anti-liver kidney microsomal 1 (LKM-1) antibodies targeting CYP2D6 self-antigen and anti-liver cytosol 1 (LC-1) antibodies targeting formimidoyltransferase cyclodeaminase (FTCD) self-antigen are associated with the more pediatric variant type 2 AIH (AIH-2).[1,4] Furthermore, anti-soluble liver antigen (SLA) (or SLA/LP) antibodies targeting SepSecS self-antigen are the only AIH-specific autoantibodies detected in both subtypes and correlate with poor overall survival.[1,5–7]

Similar to other autoimmune disorders, carrying certain HLA alleles can predispose individuals to AIH. *HLA-DRB1*03:01* and *HLA-DRB1*04:01* are the main alleles associated with AIH risk across populations.[4,8–11] However, other class I and class II HLA alleles have also been described as AIH risk factors depending on the population or disease subtype. For instance, *HLA-A*01/HLA-B*08* ancestral haplotype, often associated with *HLA-DRB1*03*, predisposes individuals of European ancestry to AIH,[4,8,9] whereas *HLA-DRB1*04:05* is a risk factor in Japanese, Korean, and Argentinian populations,[12–15] and

* Corresponding author. Address: INSERM UMR1064 – Center for Research in Transplantation and Translational Immunology, CHU Nantes – Hôtel Dieu, 30 boulevard Jean Monnet, 44093, Nantes Cedex 01, France. Tel.: +33-240087410.
E-mail address: amedee.renand@univ-nantes.fr (A. Renand).
https://doi.org/10.1016/j.jhepr.2026.101952

*HLA-DRB1*07:01* is associated with the AIH-2 pediatric subtype.[9] These findings suggest specific presentation of liver self-antigens to T cells, especially autoreactive CD4 T cells, as class II HLA predisposition and autoantibody production are strong features of AIH.

Few studies have participated in the discovery of CD4 T-cell SepSecS epitopes in AIH. Using *HLA-DRB1*03:01* transgenic mouse model and T-cell hybridoma, Mix *et al.*[16] found two HLA-DRB1*03:01-restricted immunodominant SepSecS epitopes. Using *ex vivo* peptide stimulation of peripheral blood mononuclear cells (PBMCs) from Chinese patients with AIH, Zhao *et al.* found three distinct SepSecS epitopes with a suggested HLA-DRB1*04 restriction.[17] In 2025, Kramer *et al.*[18] mapped the antigen recognition of more than 200 *ex vivo* stimulated SepSecS-specific CD4 T-cell clones from 14 AIH patients and revealed multiple SepSecS epitopes across the entire amino acid sequence, including epitopes recognized by TCR clonotypes from the liver of one AIH patient. Previously, we isolated SepSecS-specific CD4 T cells from PBMCs of AIH patients using a CD154 activation-induced marker (AIM) assay after re-stimulation with SepSecS peptides, and studied their transcriptomic signature and TCR repertoire.[19,20] Four of the 10 most expanded SepSecS-specific TCR clonotypes from one AIH patient were cloned and expressed into murine hybridoma T-cell line to determine their epitope recognition *in vitro*. Co-culture with immortalized B-cell lines (as antigen-presenting cells) and SepSecS peptides was performed, and two SepSecS epitopes, also identified in other studies,[16,18] could be identified using murine IL-2 secretion by reactive TCR cell lines.[19] However, most of the known SepSecS epitopes lack demonstrated HLA restrictions, limiting the expansion of therapeutic strategies using these SepSecS epitopes.

Here, we investigate the molecular triad involved in SepSecS epitope reactivity. Using the same strategy as in our previous study,[19] we have sequenced and reconstructed SepSecS-specific CD4 TCRαβ sequences in hybridoma T-cell lines and used them for HLA-restricted peptide recognition assays. Furthermore, HLA-epitope binding predictions and TCR clustering analysis helped to deepen the understanding of liver self-antigen reactivity during AIH. Notably, our results highlight the presentation of SepSecS epitopes by HLA-DR and non-HLA-DR molecules, providing potential targets to characterize autoreactive CD4 T cells and to develop therapeutic tools in AIH.

Patients and methods

All the eligible patients signed a written informed consent before inclusion into a biobank of samples of autoimmune liver disease patients (BIO-MAI-FOIE) maintained at the Nantes University Hospital, which obtained regulatory clearance from the biomedical research Ethics Committee (COMITE DE PROTECTION DES PERSONNES OUEST IV-NANTES CPP) and all required French Research Ministries authorizations to collect biological samples (Ministère de la Recherche, ref MESR DC-2017-2987). The MAI-FOIE cohort is registered at ClinicalTrial.gov (NCT07594990). The biobank is supported by the HEPATIMGO network promoted since 2017 (RC17_0228) by Nantes University Hospital and is a prospective multicentric collection managed by the Biological Resource Center of the CHU of Nantes. All data collected were treated confidentially and participants remained free to withdraw from the study at any time, without explanation and without prejudice to their future care. The study was granted regulatory authorization by CNIL (ref 2001209v0). All AIH patients included in this study had a simplified diagnostic score superior or equal to 6 according to the simplified scoring system for AIH of the International Autoimmune Hepatitis Group.[21,22] Sex analysis was not conducted as AIH is a rare disease and predominantly affects women.[1,21] This study was carried out in accordance with the principles of the International Council for Harmonisation (ICH) Good Clinical Practice (GCP) (as adopted in France) guidelines, which build upon the ethical codes contained in the current version of the Declaration of Helsinki, the rules and recommendations of good international (ICH) and French clinical practice (good clinical practice guidelines for biomedical research on medicinal products for human use), and the European regulations and/or national legislation and regulations on clinical trials. HLA typing was performed by the HLA laboratory at the Etablissement Français du Sang (EFS) Centre-Pays de la Loire using next-generation sequencing.

SepSecS peptides

SepSecS protein sequence used in this study has the accession number AAD33963 (422 amnio acid; aa), also previously used by Mix *et al.*,[16] which differs from the curated SepSecS sequence with the accession number Q9HD40 (501aa). Of note, the sequence used lacks the first 90 amino acids (first two exons), has an addition of 10 amino acids at the start (MSTSYGCFWR), and shows two distinct amino acid substitutions (position 398: R>K, position 452: K>N) compared with the curated sequence (Table S1). Then, 20-mer peptides overlapping by 12 amino acids and spanning the entire SepSecS sequence used were synthesized (53 peptides; Synpeptide, Shanghai, China). SepSecS peptides were annotated sequentially from p1 ($SepSecS_{1-20}$) to p53 ($SepSecS_{403-422}$) according to accession number AAD33963. Sequence differences affect p1 (first 10 amino acids), p2 (first two amino acids), p39 ($SepSecS_{305-324,319:R>K}$), p40 ($SepSecS_{313-332,319:R>K}$), p46 ($SepSecS_{361-380,373:K>N}$), and p47 ($SepSecS_{369-388,373:K>N}$). Pools of five peptides were annotated from pool #1 (p1–p5) to pool #11 (p51–53, three peptides only). Comparison with the curated sequence is provided in Table S1.

Peptide re-stimulation assay

As previously described,[19,20] 10–20 × 10^6 PBMCs (at a final concentration of 10 × 10^6/ml) were stimulated for 3–4 h at 37 °C with 1 μg/ml of synthesized 20-mer overlapping peptides spanning the entire SepSecS sequence (AAD33963, Table S1) or of $SepSecS_{73-100}$ (p10/11, for testing of epitope reactivity) in RPMI 1640 with 5% human serum and 1 μg/ml anti-CD40 (130-094-133, HB14; Miltenyi Biotec, Bergisch Gladbach, Germany). After specific peptide stimulation, PBMCs were first labeled with PE-conjugated anti-CD154 (130-113-607, 5C8; Miltenyi Biotec, Bergisch Gladbach, Germany), and CD154+ cells were then enriched using anti-PE magnetic beads (130-048-801; Miltenyi Biotec, Bergisch Gladbach, Germany) and magnetic MS column (130-042-201; Miltenyi Biotec, Bergisch Gladbach, Germany). A one-tenth fraction of non-enriched cells was saved before enrichment for frequency determination. Frequency was

calculated with the formula F = n/N, where n is the number of CD154 positive cells in the bound fraction after enrichment and N is the total number of CD4+ T cells (calculated as 10 times the number of CD4+ T cells in the one-tenth non-enriched fraction that was saved for analysis). After enrichment, cells were stained with appropriate antibodies (Table S2).

Flow cytometry and cell sorting

All antibodies used are described in Table S2. Briefly, PBMCs were incubated for 20 min with a mix of antibodies and then washed before analysis or cell sorting on BD FACSCanto™ II or BD FACSAria™ II system.

Generation of T-cell hybridomas expressing cloned TCRs

Rearranged variable and constant regions of human TCR α and β chains from sorted SepSecS-specific CD4 T cells (Table S3, in bold) were cloned in the murine 58α⁻β⁻ T-cell hybridoma,[23] expressing human CD4[24] (gift from Prof. Dr. Klaus Dornmair, Munich, Germany) as previously described.[25] The entire nucleotide sequence of each TCR (synthesized by Life Technologies,Carlsbad, CA, USA) was cloned into a pRc/RSV (Life Technologies, Carlsbad, CA, USA) stable expression vector of 5224 bp possessing a neomycin resistance gene, integrating a 2A self-cleaving peptide (P2A or T2A) between the α and β chains, as described earlier.[19] Briefly, 30 μg DNA of each construct (Gene Pulser II Electroporation system; Biorad, Hercules, CA, USA) were electroporated into 5×10^6 murine 58α⁻β⁻ T-cell hybridoma which were selected in RPMI 1640 (RPMI, 31870-025; Gibco, Grand Island, NY , USA) supplemented with 10% heat-inactivated fetal bovine serum (FBS) (S1810-500, Dutscher, Bernolsheim, France, or A5256701, Gibco, Grand Island, NY , USA), 1% Glutamine (1294808; Sigma-Aldrich, Burlington, MA, USA), 1% Penicillin-Streptomycin (Pen Strep, 15140-122; Gibco, , Grand Island, NY , USA), and with 0.5 mg/ml of G418 antibiotic (Millipore, Burlington, MA, USA) for almost 3 weeks to obtain T-cell hybridomas co-expressing murine CD3, human CD4, and recombinant TCRαβ (data not shown). TCR cell lines were sorted on BD FACSAria™ II based on the mouse CD3 expression, and murine IL-2 secretion capacity was tested using a mouse IL-2 ELISA kit (88-7024-88; Invitrogen, Carlsbad, CA, USA) (data not shown).

Epstein-Barr virus B-lymphoblastoid cell lines

Epstein-Barr virus B-lymphoblastoid cell lines (BLCL) were obtained as described earlier.[19] BLCL FEB*DR3 was derived from PBMCs of the healthy donor FEB obtained from the EFS with informed consent (Blood products transfer agreement relating to biomedical research protocol 97/5-B – DAF 03/4868) (Table S4). BLCL 1064 was derived from PBMCs of the AIH patient 01-018 (Table S4). PBMCs were *in vitro* infected by EBV-containing culture supernatant from the Marmoset B95.8 cell line purchased from the American Type Culture Collection (ATCC, Rockville, MD, USA) in the presence of 1 μg/ml cyclosporine-A. Infection was performed overnight, and the next day fresh complete medium with 1 μg/ml cyclosporine-A were added. Around 3 weeks later, B blasts from EBV-infected B lymphocytes emerged. BLCLs were then cultured in complete medium (RPMI, 10% FBS, 1% Glutamine, 1% Pen Strep).

CD4 T-cell SepSecS epitope discovery

The 17 TCR cell lines were tested for their reactivity against SepSecS peptides. Briefly, 40×10^3 TCR cell lines were cultured overnight at 37 °C in the presence of 20×10^3 immortalized BLCL FEB*DR3 or BLCL 1064 as antigen-presenting cells, with 5 μg/ml pool of five SepSecS peptides (Table S1), in complete medium (RPMI, 10% FBS, 1% Glutamine, 1% Pen Strep). Secreted murine IL-2 was measured in the supernatant using a mouse IL-2 ELISA kit (88-7024-88; Invitrogen, Carlsbad, CA, USA). Pool of peptides that were associated with positive IL-2 secretion were retested for each individual peptide (1 μg/ml) as described earlier. HLA restriction was tested by adding 10 μg/ml of anti-HLA-DR-blocking antibody (G46-6, 555809; BD Pharmingen, San Diego, CA, USA), anti-HLA-DQ-blocking antibody (SPVL3; gift from Dr. W. W. Kwok, Seattle, WA, USA), or anti-HLA-DP-blocking antibody (H266-200UG; Leinco Technologies, St. Louis, MO, USA) during co-culture. Each condition was tested in duplicate.

HLA epitope binding prediction with NetMHCIIpan 4.1 BA tool

HLA class II binding predictions were performed using the Immune Epitope Database (IEDB) analysis resource NetMHCIIpan (ver. 4.1) tool.[26] The SepSecS epitopes identified were input with their corresponding HLA alleles. Amino acid sequences were merged for epitopes including overlapping peptides. As *HLA-DQA1* alleles of patient 01-192 were unknown, the HLA query for p27 ($SepSecS_{209-228}$) epitope included all available *HLA-DQA1* alleles in combination with *HLA-DQB1*02:02*. For p47 ($SepSecS_{369-388}$) binding prediction, $SepSecS_{369-388,373:N>K}$ was also input according to the curated SepSecS sequence (Q9HD40, Table S1). Output data are summarized in Table S5, with half-maximal inhibitory concentration (IC_{50}) values and suggested binding affinity where IC_{50} <50 nM suggests high affinity (high), IC_{50} <500 nM suggests intermediate affinity (inter), IC_{50} <5,000 nM suggests low affinity (low), and IC_{50} >5,000 nM suggests no binding (no). Strong binders are shown in bold.

TCR clustering with GLIPH2 algorithm

The GLIPH2 algorithm was used for SepSecS-specific TCR clustering and prediction of HLA restriction.[27,28] TCRs sequences (CDR3β, TRBV, TRBJ, CDR3α, patient ID, frequency) from SepSecS-specific CD154 AIM assays[19,20] (Table S3) or from sorted HLA-DRB1*03:01-$SepSecS_{187-197}$ tetramer-specific CD4 T cells[20] and $SepSecS_{177-204}$-specific TCR cell lines, with known HLA alleles of patients (Table 1), were input in GLIPH2 (reference version: version 2.0, cell reference: CD4, all_aa_interchangeable: no). Output files with annotation (TCR cell lines in bold, prediction of HLA restriction in red) are presented in Tables S6 and S7. Single TCRs found in different index were merged in the index with the most significant Fisher score.

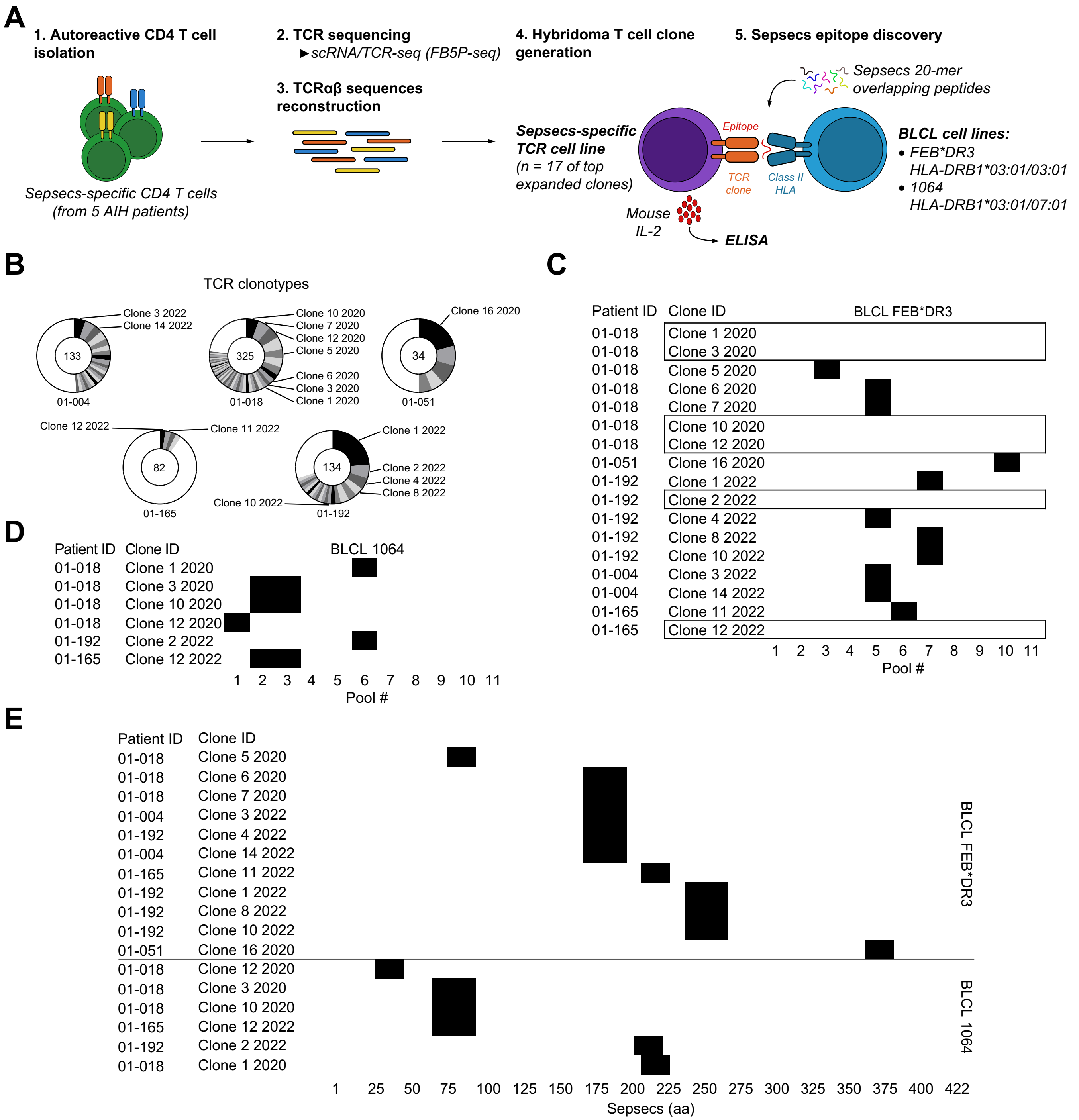

Fig. 1. TCR hybridoma cell line generation and SepSecS epitopes discovery. (A) Experimental design for SepSecS epitope discovery. (B) Distribution of the 17 TCRαβ clonotypes selected for epitope discovery (clone ## 20##) among total SepSecS-specific TCR clonotypes of each patient. Numbers indicate individual clonotypes. Colored sectors indicate expanded clonotypes (at least two cells with the same TCR). (C) Reactivity of clones against pools of SepSecS peptides when cultured with BLCL FEB*DR3. Boxes indicate non-responding clones. (D) Reactivity of boxed clones in C against pools of SepSecS peptides when cultured with BLCL 1064. (E) Reactivity of clones against individual SepSecS peptides in responding pools. Black squares indicate positive IL-2 secretion in C–E. aa, amino acid; AIH, autoimmune hepatitis; BLCL, B-lymphoblastoid cell line; HLA, human leukocyte antigen; TCR, T-cell receptor.

Statistical analysis

Statistical comparisons were performed using the GraphPad Prism software V.6 (GraphPad Software, La Jolla, CA, USA). A p value <0.05 was considered significant. Sample size represents the maximum number of samples obtained during the time of the study, as anti-SLA positive AIH patients are rare (*DP*2.1* anti-SLA positive AIH patients even more) .

Table 1. Age, sex, class II HLA genotypes of AIH patients, and scRNA-seq data accession.[19,20]

Patient ID	Age	Sex	DRB1*	DQB1*	DPA1*	DPB1*	Ref. RNA-seq	NCBI GEO numbers	SepSecS TCR cell lines (n)
01-001	49	F	03:01/16:01	02:01/05:02	02:01/02:01	01:01/10:01	NA	NA	
01-004	18	F	03:01/03:01	02:01/02:01	01:03/02:01	01:01/04:01	Renand *et al.*[19] and Cardon *et al.*[20]	GSE149298, GSE270739	Yes (2)
01-018	21	F	03:01/07:01	02:01/02:02	01:03/02:01	01:01/04:01	Renand *et al.*[19] and Cardon *et al.*[20]	GSE149298, GSE270739	Yes (7)
01-028	19	M	01:01/10:01	05:01/05:01	01:03/01:03	04:01/06:01	Renand *et al.*[19] and Cardon *et al.*[20]	GSE149298, GSE270739	
01-046	46	F	03:01/03:01	02:01/02:01	01:03/02:01	01:01/04:01	NA	NA	
01-051	8	F	03:01/03:01	02:01/02:01	01:03/01:03	03:01/06:01	Renand *et al.*[19]	GSE149298	Yes (1)
01-128	16	F	03:01/03:01	02:01/02:01	01:03/02:01	01:01/04:01	Renand *et al.*[19]	GSE149298	
01-140	14	F	03:01/07:01	02:01/02:02	01:03/02:02	04:01/05:01	Cardon *et al.*[20]	GSE270739	
01-165	29	F	03:01/03:01	02:01/02:01	02:01/02:01	01:01/01:01	Cardon *et al.*[20]	GSE270739	Yes (2)
01-192	18	F	03:01/07:01	02:01/02:02	NA	NA	Cardon *et al.*[20]	GSE270739	Yes (5)
01-354	59	F	03:01/03:01	02:01/02:01	NA	02:02/04:01	NA	NA	
05-001	48	F	04:01/13:01	03:02/06:03	NA	04:01/04:01	Cardon *et al.*[20]	GSE270739	
05-004	37	F	03:01/01:01	02:01/05:01	NA	02:02/04:01	NA	NA	
07-009	53	F	04:01/15:03	03:02/06:02	NA	02:01/13:01	NA	NA	
08-002	42	F	07:01/13:01	02:02/06:03	NA	02:01/04:01	NA	NA	

All the patients from whom TCR cell lines were derived carry the *HLA-DRB1*03:01* allele. Age: age at diagnosis; AIH, autoimmune hepatitis; F, female; HLA, human leukocyte antigen; M, male; NA, no data available; scRNA-seq. single-cell RNA sequence; TCR, T-cell receptor.

Results

Epitope discovery of the most expanded SepSecS-specific CD4 T-cell clones in AIH patients

In this study, we cloned and expressed 17 SepSecS-specific CD4 T-cell clones (clone defined as more than two cells with the same TCRαβ sequences) into murine hybridoma T-cell line, including the four clones studied previously.[19] These 17 clones were derived from blood CD4 T cells that upregulated CD154 after *ex vivo* re-stimulation with SepSecS peptides from five AIH patients in our previous studies, and represented 18% to 57% of the SepSecS-specific CD4 T clones identified at the single cell level[19,20] (Fig. 1A, B, Tables 1 and 2, and Table S3). After *in vitro* validation that the TCRs were well transfected and expressed at the plasma membrane, by using anti-CD3 antibody stimulation (data not shown), each TCR cell line (clone ## 20##) was tested for its reactivity against SepSecS peptide pools using murine IL-2 secretion (Fig. 1A). The SepSecS protein (see Patients and methods) was covered by 20-mer overlapping peptides and each pool contained five peptides (Table S1). All the TCR cell lines were derived from *HLA-DRB1*03:01/03:01* homozygous patients or *HLA-DRB1*03:01/07:01* heterozygous patients (Table 1). Thus, TCR cell lines were first tested in the presence of *HLA-DRB1*03:01/03:01* homozygous immortalized B-cell lines (BLCL FEB*DR3) as antigen-presenting cells (Fig. 1A, C and Table S4). Of the 17 TCRs, 11 reacted against SepSecS peptides presented by BLCL FEB*DR3 (Fig. 1C). The data revealed that five distinct TCRs from three different patients reacted against peptides included in pool #5. Three TCRs from the same patient (01-192) reacted against peptides in pool #7. The three other TCRs reacted against peptides from pool #3, #6, or #10 (Fig. 1C).

The six non-responding TCRs were re-tested in the presence of *HLA-DRB1*03:01/07:01* heterozygous immortalized B-cell lines (BLCL 1064) as antigen-presenting cells (Fig. 1A, D and Table S4). Three distinct TCRs from two different patients reacted against peptides in pool #2 and #3. Two TCRs from two different patients reacted against peptides in pool #6. The last TCR reacted against peptides in pool #1. These data suggest that these six TCRs reacted against SepSecS peptides with a different HLA restriction than the other 11 TCRs tested (Fig. 1C).

Next, we identified the specific epitope recognized by each TCR cell line. We tested individually each of the five peptides present in the reacting pools and measured the murine IL-2 secretion (Fig. 1E). Starting with the TCRs that reacted against peptides in pools presented by BLCL FEB*DR3, the five TCRs reacting against pool #5 recognized specifically peptides 23 and 24 (p23/24; $SepSecS_{177-204}$) (Fig. 1E and Table 2). The three TCRs from the patient 01-192 which reacted against peptides in pool #7 recognized specifically peptides 32 and 33 (p32/33; $SepSecS_{249-276}$). The three other TCRs recognized specifically peptides 11 (p11; $SepSecS_{81-100}$), 28 (p28; $SepSecS_{217-236}$), and 47 (p47; $SepSecS_{369-388}$) (Fig. 1E and Table 2). Regarding TCRs that reacted against peptides in pools presented by BLCL 1064, the three TCRs reacting against pool #2 and #3 recognized specifically peptides 10 and 11 (p10/11; $SepSecS_{73-100}$) (Fig. 1E and Table 2). Among the two TCRs reacting against peptides from pool #6, one recognized specifically peptide 27 (p27; $SepSecS_{209-228}$) and the other one recognized specifically peptide 28 (p28;

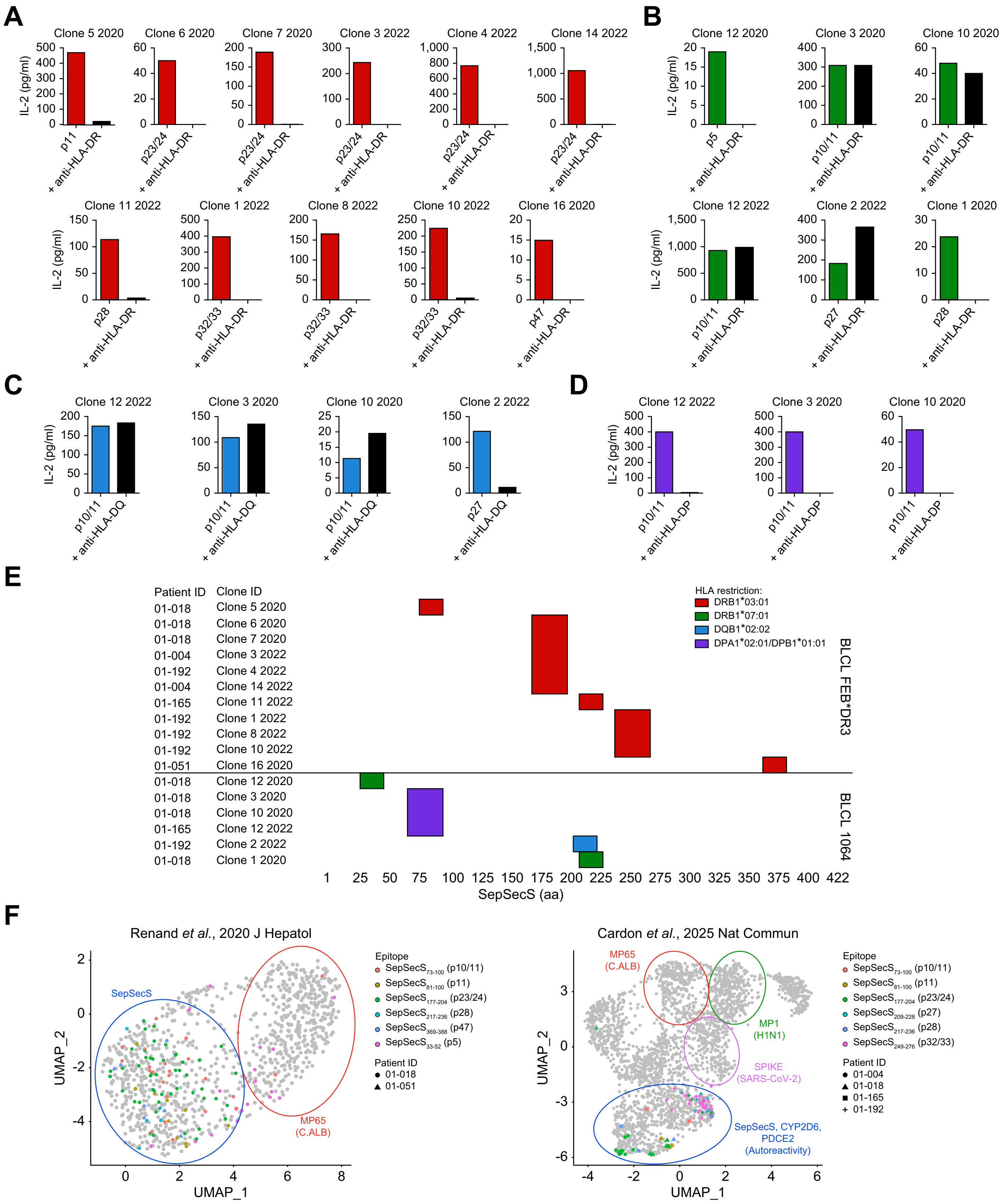

Fig. 2. Class II HLA restriction of identified CD4 T-cell SepSecS epitopes. (A and B) Epitope reactivity of clones with anti-HLA-DR-blocking antibody and BLCL FEB*DR3 (A) or BLCL 1064 (B). (C) Epitope reactivity of clones still responding in B with anti-HLA-DQ-blocking antibody. (D) Epitope reactivity of clones still responding in C with anti-HLA-DP-blocking antibody. (E) Mapping of SepSecS epitope reactivity colored by class II HLA restriction. (F) UMAP representations of single-cell transcriptomes of antigen-specific CD4 T cells from previous studies[19,20] colored by SepSecS epitope reactivity and patient ID. Circles annotate overall antigen-specific signatures: SepSecS; MP65 from *Candida albicans* (C.ALB); autoreactivity (SepSecS, CYP2D6, PDCE2); MP1 from H1N1 virus, SPIKE from SARS-CoV-2 virus. aa, amino acid; BLCL, B-lymphoblastoid cell line; HLA, human leukocyte antigen; UMAP, uniform manifold approximation and projection.

A

Group	TCR cell line	SepSecS epitope	Index	Pattern	CDR3β	Vβ	Jβ	CDR3α	Patient ID	Pred. HLA restriction
I			1	SLQ	CASSLQGADSDTQYF	TRBV5-1	TRBJ2-3	CAVMGGSNYKLTF	01-004	DPA1*02:01/DPB1*01:01
			1	SLQ	CASSLQGGDTDTQYF	TRBV5-1	TRBJ2-3	CAVMGASDYKLSF	01-004	DPA1*02:01/DPB1*01:01
			$1_{18,26,35}$	SLQ	CASSLQVGGETQYF	TRBV7-2	TRBJ2-5	CAVSLPTGGGNKLTF	01-018	DPA1*02:01/DPB1*01:01
			$1_{4,14,28}$	SLQ	CASSLQAGGPDTQYF	TRBV7-2	TRBJ2-3	N.A.	01-018	DPA1*02:01/DPB1*01:01
			$1_{26,35}$	SLQ	CASSLQVDTKSTQYF	TRBV7-2	TRBJ2-5	N.A.	01-018	DPA1*02:01/DPB1*01:01
			1	SLQ	CASSLQGTDTQYF	TRBV7-2	TRBJ2-3	N.A.	01-018	DPA1*02:01/DPB1*01:01
			$1_{4,14,21}$	SLQ	CASSLQASGTDTQYF	TRBV7-2	TRBJ2-3	CALSDGVTGGGNKLTF	01-018	DPA1*02:01/DPB1*01:01
			1	SLQ	CASSLQGDTDTQYF	TRBV12-3	TRBJ2-3	N.A.	01-018	DPA1*02:01/DPB1*01:01
			1	SLQ	CASSLQPGDTQYF	TRBV7-3	TRBJ2-3	N.A.	01-018	DPA1*02:01/DPB1*01:01
			1	SLQ	CASSLQQGASDTQYF	TRBV7-2	TRBJ2-3	N.A.	01-018	DPA1*02:01/DPB1*01:01
			$1_{4,14,21,28}$	SLQ	CASSLQAGGTDTQYF	TRBV7-3	TRBJ2-3	N.A.	01-018	DPA1*02:01/DPB1*01:01
			$1_{6,13,31}$	SLQ	CASSLQTGTDTQYF	TRBV7-2	TRBJ2-3	N.A.	01-018	DPA1*02:01/DPB1*01:01
			$1_{6,13,31}$	SLQ	CASSLQTGDYGYTF	TRBV7-2	TRBJ1-2	N.A.	01-018	DPA1*02:01/DPB1*01:01
			1	SLQ	CASSLQLAAINEQFF	TRBV7-2	TRBJ2-1	CALSTGYGGSQGNLIF	01-018	DPA1*02:01/DPB1*01:01
			$1_{4,14,28}$	SLQ	CASSLQAGFTDTQYF	TRBV7-2	TRBJ2-3	CAVEDPGSARQLTF	01-018	DPA1*02:01/DPB1*01:01
			1	SLQ	CASSLQGSSYNEQFF	TRBV7-2	TRBJ2-1	N.A.	01-128	DPA1*02:01/DPB1*01:01
			1	SLQ	CASSSLQGTTVDEQFF	TRBV11-2	TRBJ2-1	N.A.	01-128	DPA1*02:01/DPB1*01:01
			$1_{6,13}$	SLQ	CASSLQTSGGTDTQYF	TRBV7-2	TRBJ2-3	N.A.	01-128	DPA1*02:01/DPB1*01:01
			$1_{6,13,18,31}$	SLQ	CASSLQTGGETQYF	TRBV7-2	TRBJ2-5	N.A.	01-128	DPA1*02:01/DPB1*01:01
			$1_{26,35}$	SLQ	CASSLQVSDTGELFF	TRBV7-2	TRBJ2-2	N.A.	01-128	DPA1*02:01/DPB1*01:01
			$1_{6,13}$	SLQ	CASSLQTSAHNEQFF	TRBV7-2	TRBJ2-1	CAVGRGGADGLTF	01-165	DPA1*02:01/DPB1*01:01
	Clone 12 2022	$SepSecS_{73-100}$ (p10/11)	1	SLQ	CASSLQRAVQETQYF	TRBV7-2	TRBJ2-5	CAFMTQNYGGATNKLIF	01-165	DPA1*02:01/DPB1*01:01
			$1_{4,14}$	SLQ	CASSLQAATNEKLFF	TRBV7-2	TRBJ1-4	CAGRPAGSARQLTF	01-165	DPA1*02:01/DPB1*01:01
II			2_{8}	PQTG	CASSQPQTGGPDTQYF	TRBV6-8	TRBJ2-3	CAVSESLTGGGNKLTF	01-004	DPB1*01:01
			$2_{8,11}$	PQTG	CASSPQTGGDYNEQFF	TRBV28	TRBJ2-1	N.A.	01-018	DPB1*01:01
	Clone 3 2020	$SepSecS_{73-100}$ (p10/11)	$2_{8,11}$	PQTG	CASSPQTGVQETQYF	TRBV7-2	TRBJ2-5	CAHYMNYGGSQGNLIF	01-018	DPB1*01:01
			$2_{8,11}$	PQTG	CASSPQTGANEKLFF	TRBV7-2	TRBJ1-4	N.A.	01-018	DPB1*01:01
			$2_{8,11}$	PQTG	CASSPQTGENEKLFF	TRBV7-2	TRBJ1-4	N.A.	01-018	DPB1*01:01
			$2_{8,11}$	PQTG	CASSPQTGTDTQYF	TRBV7-2	TRBJ2-3	N.A.	01-128	DPB1*01:01
III			23	GVYG	CASSQSRQGVYGYTF	TRBV3-1	TRBJ1-2	CAVGAGGYNKLIF	01-018	DRB1*07:01
			23	GVYG	CASSFPGQGVYGYTF	TRBV7-2	TRBJ1-2	CAVRDIGGGYQKVTF	01-018	DRB1*07:01
	Clone 6 2020	$SepSecS_{177-204}$ (p23/24)	23	GVYG	CASSFPGQGVYGYTF	TRBV7-2	TRBJ1-2	CAVGSGGYNKLIF	01-018	DRB1*07:01
			23	GVYG	CASSSPQGGVYGYTF	TRBV7-8	TRBJ1-2	N.A.	01-192	DRB1*07:01
			23	GVYG	CATSSHTQGVYGYTF	TRBV15	TRBJ1-2	N.A.	01-192	DRB1*07:01
IV			34	SLGL%YE	CASSLGLNYEQYF	TRBV11-2	TRBJ2-7	CAVQGGSEKLVF	01-004	N.A.
	Clone 8 2022	$SepSecS_{249-276}$ (p32/33)	34	SLGL%YE	CASSLGLSYEQYF	TRBV11-2	TRBJ2-7	CAVQGGSEKLVF	01-192	N.A.
V	Clone 3 2022	$SepSecS_{177-204}$ (p23/24)	159	SLVGGSSYNE	CASSLVGGSSYNEQFF	TRBV11-2	TRBJ2-1	CAVGAAGTASKLTF	01-004	N.A.
			159	SLVGGSSYNE	CASSLVGGSSYNEQFF	TRBV11-3	TRBJ2-1	CAVGAAGTASKLTF	01-004	N.A.
VI	Clone 7 2020	$SepSecS_{177-204}$ (p23/24)	297	SSFDRGNYG	CASSSFDRGNYGYTF	TRBV7-3	TRBJ1-2	CAVTPSGGYQKVTF	01-018	N.A.
			297	SSFDRGNYG	CASSSFDRGNYGYTF	TRBV7-3	TRBJ1-2	CAGPETSYDKVIF	01-018	N.A.

"X_Y": X principal index (red pattern), Y additonal index (blue pattern)

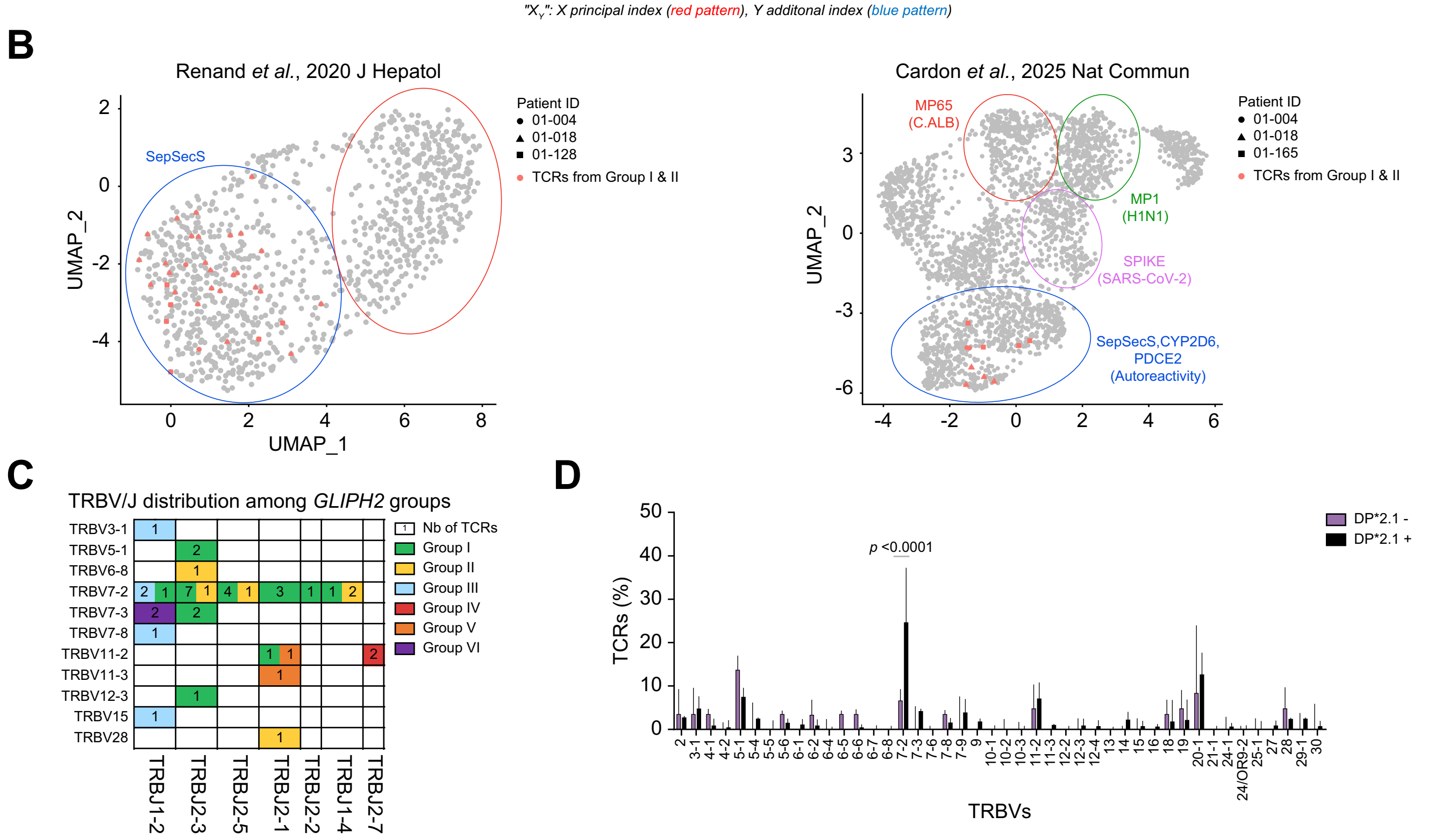

Fig. 3. SepSecS-specific TCR clustering using GLIPH2 algorithm. (A) SepSecS-specific TCR groups of interest each containing one clone tested for epitope reactivity. (B) UMAP representations of single-cell transcriptomes of antigen-specific CD4 T cells from previous studies[19,20] colored by TCRs from groups I and II and patient ID. Circles annotate overall antigen-specific signatures (see Fig. 1F). (C) Matrix of *TRBV* and *TRBJ* usage in TCR groups. (D) *TRBV* proportion within SepSecS-specific TCRs from AIH patients carrying *HLA-DPA1*02:01~HLA-DPB1*01:01* (*DP*2.1*) haplotype (n = 4, black) or not (n = 5, purple). Data are presented as median ± interquartile range in D. Sidak's multiple comparisons test was used in D. Significant adjusted *p* value is indicated. C.ALB, *Candida albicans*; HLA, human leukocyte antigen; TCR, T-cell receptor; UMAP, uniform manifold approximation and projection.

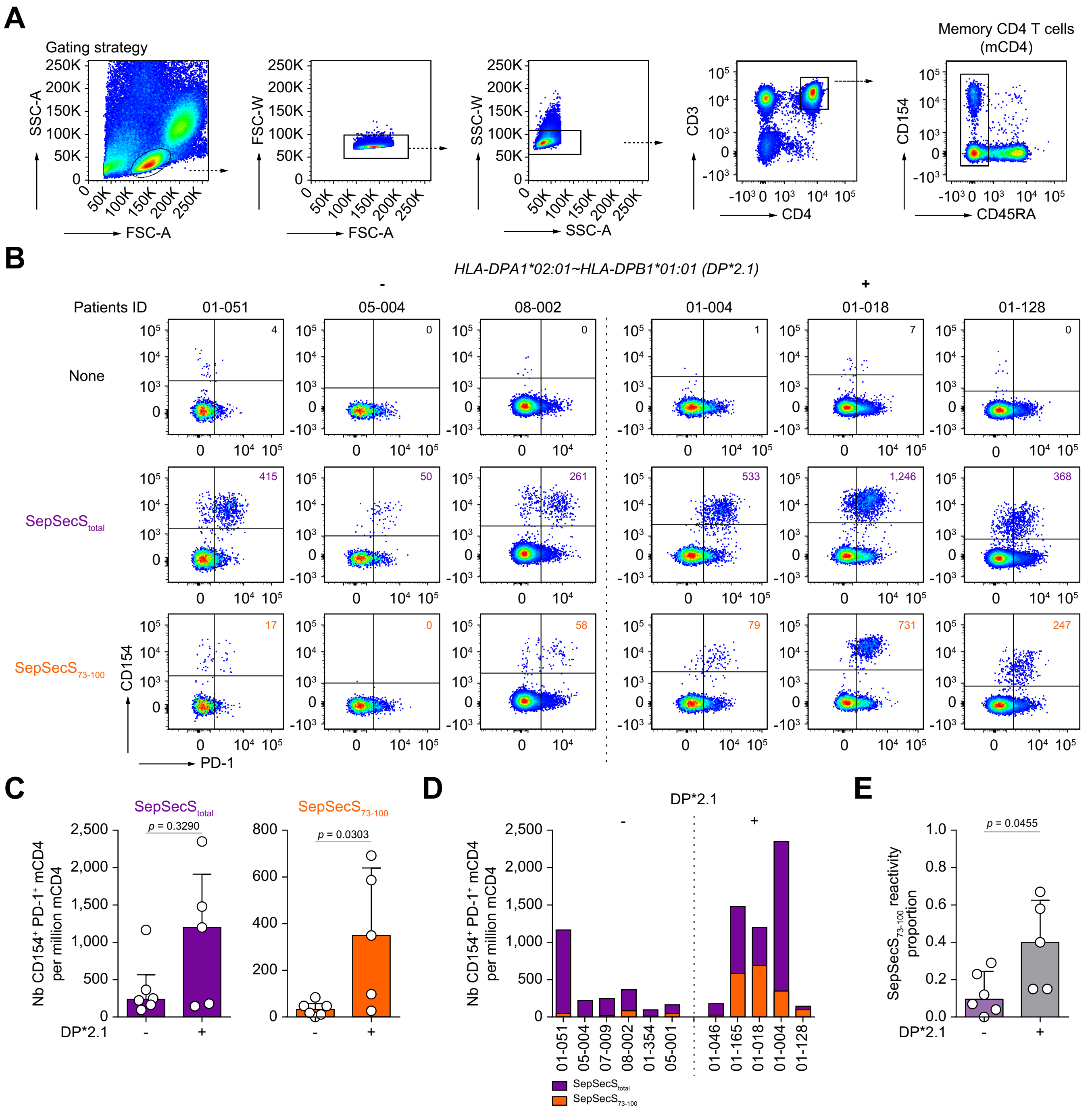

Fig. 4. SepSecS$_{73-100}$ epitope represents an important proportion of SepSecS CD4 T-cell reactivity in *HLA-DPA1*02:01~HLA-DPB1*01:01* AIH patients. (A) Gating strategy for CD45RA-memory CD4 T cells (mCD4) in PBMCs. (B) Representative pseudo color dot plots of mCD4 reacting against (CD154+ PD-1+) total SepSecS peptides (SepSecS$_{total}$; purple) or SepSecS$_{73-100}$ peptides (orange) in *DP*2.1*-positive (n = 5) and *DP*2.1*-negative (n = 6) AIH patients. (C) Frequency of SepSecS$_{total}$-reactive and SepSecS$_{73-100}$-reactive mCD4 per million mCD4 in both groups. (D) Frequency of SepSecS$_{73-100}$-reactive mCD4 (orange) stacked on SepSecS$_{total}$-reactive mCD4 (purple) for each patient. (E) SepSecS$_{73-100}$ reactivity proportion within total SepSecS reactivity in *DP*2.1*-positive and *DP*2.1*-negative AIH patients. Data are presented as median ± interquartile range in C and E. Two-sided Mann-Whitney test was used in C and E; *p* values are indicated. AIH, autoimmune hepatitis; PBMCs, peripheral blood mononuclear cells.

SepSecS$_{217-236}$) (Fig. 1E and Table 2). The last TCR recognized specifically peptide 5 (p5; SepSecS$_{33-52}$) from pool #1.

These data demonstrate that SepSecS-specific CD4 T cells from AIH patients recognize distinct T-cell epitopes (eight distinct epitopes recognized by 17 clones) with probable distinct HLA restriction. Interestingly, three of the epitopes described in this study were recognized by more than two T-cell clones, suggesting strong immunogenicity (p10/11: SepSecS$_{73-100}$; p23/24: SepSecS$_{177-204}$; p32/33: SepSecS$_{249-276}$).

Table 2. TCR cell lines and identified class II HLA-restricted SepSecS epitopes.

Patient ID	Freq	TRAV	TRAJ	CDR3α	TRBV	TRBJ	CDR3β	TCR cell line ID	Epitope	SepSecS region	HLA restriction	GLIPH2 index
01-004	7	TRAV8-3	TRAJ44	CAV-GAAGTASKLTF	TRBV11-2	TRBJ2-1	CASSLVGGSSY-NEQFF	clone 3 2022	p23/24	177-204	DRB1*03:01	159
01-004	5	TRAV8-3	TRAJ6	CAV-GSSGGSYIPTF	TRBV11-2	TRBJ2-2	CASSLGLSTNTGELFF	clone 14 2022	p23/24	177-204	DRB1*03:01	NA
01-018	18	TRAV19	TRAJ42	CAL-SEAGGSQGNLIF	TRBV20-1	TRBJ1-1	CSASRGQGLVN-TEAFF	clone 10 2020	p10/11	73-100	DPA1*02:01/ DPB1*01:01	NA
01-018	18	TRAV38-2/DV8	TRAJ58	CAYRSA-LISGSRLTF	TRBV2	TRBJ2-5	CASSEKNLAWETQYF	clone 12 2020	p5	33-52	DRB1*07:01	NA
01-018	18	TRAV8-1	TRAJ13	CAV-TPSGGYQKVTF	TRBV7-3	TRBJ1-2	CASSSFDRGNYGYTF	clone 7 2020	p23/24	177-204	DRB1*03:01	297
01-018	13	TRAV21	TRAJ11	CAMSQDRNS-GYSTLTF	TRBV18	TRBJ2-3	CASSPAGAADTQYF	clone 5 2020	p11	81-100	DRB1*03:01	NA
01-018	7	TRAV8-3	TRAJ4	CAVGSGGYNKLIF	TRBV7-2	TRBJ1-2	CASSFPGQGVYGYTF	clone 6 2020	p23/24	177-204	DRB1*03:01	23
01-018	6	TRAV21	TRAJ48	CAAPAAGNEKLTF	TRBV20-1	TRBJ2-3	CSAGDRTGDTDTQYF	clone 1 2020	p28	217-236	DRB1*07:01	NA
01-018	6	TRAV9-2	TRAJ42	CAHYM-NYGGSQGNLIF	TRBV7-2	TRBJ2-5	CASSPQTGVQETQYF	clone 3 2020	p10/11	73-100	DPA1*02:01/ DPB1*01:01	2,8,11
01-051	7	TRAV8-4	TRAJ15	CAV-SGNQAGTALIF	TRBV4-1	TRBJ2-5	CASSQGGTSG-GLYQETQYF	clone 16 2020	p47	369-388	DRB1*03:01	NA
01-165	2	TRAV24	TRAJ37	CAIPFGNTGKLIF	TRBV5-6	TRBJ2-5	CASSLWTSGGVWET-QYF	clone 11 2022	p28	217-236	DRB1*03:01	NA
01-165	2	TRAV38-1	TRAJ32	CAFMTQNYG-GATNKLIF	TRBV7-2	TRBJ2-5	CASSLQRAVQETQYF	clone 12 2022	p10/11	73-100	DPA1*02:01/ DPB1*01:01	1
01-192	32	TRAV12-2	TRAJ33	CAVNDSNYQLIW	TRBV5-4	TRBJ2-3	CASSLAGLSSTDT-QYF	clone 1 2022	p32/33	249-276	DRB1*03:01	NA
01-192	8	TRAV16	TRAJ12	CALMDSSYKLIF	TRBV5-1	TRBJ1-6	CASSIGQGDSPLHF	clone 2 2022	p27	209-228	DQB1*02:02	NA
01-192	7	TRAV8-3	TRAJ44	CAV-VGNTGTASKLTF	TRBV5-4	TRBJ2-6	CASSSFGGPS-GANVLTF	clone 4 2022	p23/24	177-204	DRB1*03:01	NA
01-192	5	TRAV21	TRAJ57	CAVQGGSEKLVF	TRBV11-2	TRBJ2-7	CASSLGLSYEQYF	clone 8 2022	p32/33	249-276	DRB1*03:01	34
01-192	2	TRAV13-2	TRAJ45	CAE-TYSGGGADGLTF	TRBV5-4	TRBJ2-5	CASKLALRETQYF	clone 10 2022	p32/33	249-276	DRB1*03:01	NA

Seventeen TCR cell lines from the most expanded SepSecS-specific CD4 T cells in five AIH patients recognized eight different SepSecS epitopes with four distinct class II HLA restrictions. Only six clones were grouped with other SepSecS-specific TCRs in the GLIPH2 analysis.

AIH, autoimmune hepatitis; Freq, cell number (frequency of clone); HLA, human leukocyte antigen; NA, no data available; TCR, T-cell receptor.

Autoreactive TCRs recognize SepSecS epitopes presented by HLA-DR, HLA-DQ, and HLA-DP complexes

Next, we attempted to determine the class II HLA restriction of the identified epitopes (Fig. 2). Each TCR cell line was cultured in the presence of BLCL FEB*DR3 (Fig. 2A) or BLCL 1064 (Fig. 2B), and specific peptide(s) with or without anti-HLA-DR-blocking antibody. All the TCRs recognizing a peptide presented by BLCL FEB*DR3 were restricted to HLA-DR (Fig. 2A, E). Indeed, the use of anti-HLA-DR-blocking antibody inhibited IL-2 production by TCR cell lines in culture with BLCL FEB*DR3 and the specific peptide(s). Because BLCL FEB*DR3 was an *HLA-DRB1*03:01/03:01* homozygous immortalized B-cell line, we concluded that these tested TCRs were specific of the corresponding peptide presented by HLA-DRB1*03:01 (Fig. 2E and Table S4). Among them, five distinct TCRs (clone 3 2022, clone 14 2022, clone 6 2020, clone 7 2020, and clone 4 2022) recognized the HLA-DRB1*03:01-restricted SepSecS$_{177-204}$ epitope (p23/24). Three distinct TCRs (clone 1 2022, clone 8 2022, and clone 10 2022) from the same patient (01-192) recognized the HLA-DRB1*03:01-restricted SepSecS$_{249-276}$ epitope (p32/33). The HLA-DRB1*03:01-restricted SepSecS$_{81-100}$ (p11), SepSecS$_{217-236}$ (p28), and SepSecS$_{369-388}$ (p47) epitopes were recognized by unique TCRs (clone 5 2020, clone 11 2022, and clone 16 2020, respectively; Fig. 2A, E). Interestingly, SepSecS$_{184-198}$ (similar to p23/24) and SepSecS$_{373-386}$ (similar to p47) were already described as immunodominant HLA-DRB1*03:01-restricted epitopes,[16] which validate our approach in this study.

We then determined the HLA restriction of the TCRs that could not recognize peptides presented by BLCL FEB*DR3 but recognized those presented by BLCL 1064, an *HLA-DRB1*03:01/07:01* heterozygous immortalized B-cell line (Fig. 2B). The use of an anti-HLA-DR-blocking antibody inhibited the production of IL-2 only for two TCR cell lines (clone 12 2020 and clone 1 2020), suggesting that SepSecS$_{33-52}$ (p5) and SepSecS$_{217-236}$ (p28) were HLA-DRB1*07:01-restricted epitopes (Fig. 2B, E). The four other TCR cell lines still produced IL-2 in the presence of anti-HLA-DR-blocking antibody, suggesting that these were HLA-DQ- or HLA-DP-restricted epitopes (Fig. 2B). The use of an anti-HLA-DQ-blocking antibody inhibited IL-2 production for one TCR cell line (clone 2 2022), suggesting that SepSecS$_{209-228}$ (p27) was HLA-DQB1*02:02-restricted epitope, as this epitope was not presented by the *HLA-DQB1*02:01/02:01* homozygous BLCL FEB*DR3 cell line (Fig. 2C, E and Table S4). The three other TCR cell lines (clone 12 2022, clone 3 2020, and clone 10 2020) were still responsive to the SepSecS$_{73-100}$ epitope with anti-HLA-DR- or anti-HLA-DQ-blocking antibodies, suggesting HLA-DP restriction (Fig. 2B, C). The use of anti-HLA-DP-blocking antibody confirmed the HLA-DP restriction of this epitope by inhibiting the production of IL-2 for the three TCR cell lines (Fig. 2D). The analysis of *HLA-DP* genotypes between BLCL cell lines and AIH patients from whom reactive TCR cell lines were derived revealed that the SepSecS$_{73-100}$ (p10/11) epitope is an HLA-DPA1*02:01/HLA-DPB1*01:01-restricted epitope, as the corresponding alleles are only shared by both patients (01-018 and 01-165) and BLCL 1064 cell line, and not by BLCL FEB*DR3 cell line (Fig. 2E, Table 1, and Table S4). The suggested class II HLA restriction of each identified SepSecS epitope was in line with the HLA genotypes of AIH patients from whom TCR cell lines were generated (Tables 1 and 2). However, epitope binding prediction analysis using the NetMHCIIpan 4.1 BA tool[26] revealed that only the SepSecS$_{33-52}$ (p5) epitope was a strong binder for its corresponding class II HLA complex, HLA-DRB1*07:01 (Table S5).

Mapping of selected TCR clonotypes and their SepSecS epitope recognition on uniform manifold approximation and projection (UMAP) representations of single-cell transcriptomes of antigen-specific CD4 T cells from the previous studies[19,20] showed that reactive clones are found mostly in groups of cells with SepSecS-specific and/or autoreactive (SepSecS and CYP2D6 targeted in AIH, PDCE2 targeted in primary biliary cholangitis) transcriptomic signature (Fig. 2F and Fig. S1). These cells were distinguished from cells reactive for foreign antigens (MP65 from *Candida albicans* [C.ALB]; MP1 from influenza virus [H1N1]; SPIKE from SARS-CoV-2 virus) by their exhausted profile (*CTLA4*, *TIGIT*) and B-helper cytokine expression[19,20] (*IL21*, *CXCL13*) (Fig. S1). The only exception is the TCR clonotype reacting against the HLA-DRB1*07:01-restricted SepSecS$_{33-52}$ (p5) epitope that comprised many single cells outside of the overall SepSecS reactivity profile (Fig. 2F). The scattered distribution of selected TCR clonotypes within the overall SepSecS/autoreactive signatures suggests an influence from donors rather than epitope reactivity or class II HLA restriction[19,20] (Fig. 2F).

These results highlighted multiple SepSecS sequences recognized by autoreactive TCRs from AIH patients, with HLA-DR, HLA-DQ, or HLA-DP restrictions. While we confirmed the HLA-DRB1*03:01 restriction of the previously described SepSecS$_{177-204}$ (p23/24) and SepSecS$_{369-388}$ (p47) epitopes,[16,19,20] we uncovered a new epitope with the HLA-DPA1*02:01/HLA-DPB1*01:01-restricted SepSecS$_{73-100}$ epitope recognized by three distinct TCRs from two AIH patients.

GLIPH2 algorithm suggests that SepSecS$_{73-100}$ epitope is recognized by multiple TCRs from distinct AIH patients

We identified SepSecS epitopes and their HLA restriction with TCR cell lines derived from the most expanded clones in five AIH patients, so we investigated if other autoreactive TCRs could recognize these epitopes. We used GLIPH2 algorithm to group TCRs that are predicted to bind the same HLA-restricted epitope according to the similarity of TRBV/J segments and CDR3 motifs.[27,28] To support the concept of common epitope recognition by autoreactive TCRs from a unique CDR3 motif-based GLIPH2 group, we input the five TCRs from clones that recognized the HLA-DRB1*03:01-restricted SepSecS$_{177-204}$ epitope (p23/24) *in vitro* (Table 2) and TCR clonotypes of sorted SepSecS$_{187-197}$-loaded HLA-DRB1*03:01-tetramer-specific CD4 T cells from six HLA-DRB1*03:01-positive AIH patients[20] (including 01-004 and 01-018 patients), and we ran the algorithm (Table S6). The TCR cluster with the most significant Fisher score comprised nine TCRs with a GVYG-motif from three patients, including the TCR from clone 6 2020. This illustrates that among 60 tetramer-specific TCR clonotypes, eight of them (13.3%) share a CDR3 motif with a TCR cell line that reacted against the same epitope *in vitro*, suggesting that the proximity of CDR3 motifs between autoreactive TCRs could be linked to a common self-epitope reactivity (Table S6).

Thus, to determine if epitopes identified *in vitro* could be recognized by other TCRs, we ran GLIPH2 algorithm on 484 distinct SepSecS-specific TCRs isolated from nine AIH patients using the CD154 AIM assay, including the 17 TCRs used for epitope discovery[19,20] (Tables 1 and 2 and Table S3). By this technique, we identified six groups of TCRs of interest, each containing one TCR with a SepSecS epitope recognition *in vitro* (Fig. 3A and Table S7). Group I comprised 23 TCRs with an SLQ motif from four patients, including the TCR from clone 12 2022, and represented the cluster of TCRs with the most significant Fisher score. Group II comprised six TCRs with a PQTG motif from three patients, including the TCR from clone 3 2020, and had the second most significant Fisher score. Group III comprised five TCRs with a GVYG motif from two patients, including the TCR from clone 6 2020. Group IV comprised two TCRs from two patients, including the TCR from clone 8 2022. Finally, groups V and VI each comprised two TCRs with the exact same CDR3β amino acid sequence (and CDR3α amino acid sequence for group V) from the same patient, including clone 3 2022 and clone 7 2020, respectively. However, these two groups were not significantly enriched in our dataset compared with the reference TCR set (Fig. 3A and Table S7).

Among the 17 TCRs used for epitope discovery *in vitro*, only six of them were clustered with distinct TCR clonotypes. Notably, TCRs from clone 12 2022 and clone 3 2020 (groups I and II, respectively) that recognized the $SepSecS_{73-100}$ (p10/11) epitope were clustered with 27 other TCRs from four patients, suggesting similar epitope recognition despite variable CDR3 motifs (Fig. 3A). Furthermore, *HLA-DPA1*02:01* and/or *HLA-DPB1*01:01* alleles were enriched in both groups, which supports the determined HLA restriction of the $SepSecS_{73-100}$ epitope (Fig. 2E and Fig. 3A). However, the third clone that recognized this epitope (clone 10 2020) was not clustered in these groups as it lacks both the enriched amino acid motifs (Fig. 3A, Table 2, and Table S7). Similarly, TCR clones that recognized the $SepSecS_{177-204}$ epitope (p23/24) or the $SepSecS_{249-276}$ (p32/33) epitope *in vitro* were not clustered together, as their CDR3 motifs were not shared. Nonetheless, TCRs from group III share a GVYG motif, also found in multiple tetramer-specific TCRs as shown previously, which supports the prediction of epitope reactivity (Fig. 3A, Table S6, and Table S7). However, GLIPH2 algorithm predicted HLA-DRB1*07:01 restriction for TCRs from group III, whereas we demonstrated that clone 6 2020 recognized the $SepSecS_{177-204}$ epitope with HLA-DRB1*03:01 restriction, which is consistent with previous studies[16,20] (Fig. 2E, Fig. 3A, Table 2, and Table S7). This may be explained by the fact that GLIPH2 algorithm suggests HLA restriction according to the enrichment of HLA alleles from index contributors. Consequently, this could bias the prediction of HLA-DRB1*03:01 restriction, as this allele is largely represented in the dataset; n = 7/9 patients *vs*. n = 3/9 patients for *HLA-DRB1*07:01* allele (Fig. 3A and Tables 1 and 2). Furthermore, in group IV, both TCRs use TRAV21, TRAJ57, TRBV11-2, and TRBJ2-7, share the exact same CDR3α amino acid sequence, and show only one amino acid difference in their CDR3β, suggesting that they could belong to a public meta-clonotype for $SepSecS_{249-276}$ (p32/33) epitope[29] (Fig. 3A, Table 2, and Table S7). Mapping of TCRs from groups I and II on transcriptomes of antigen-specific CD4 T cells from the previous studies[19,20] highlighted their SepSecS/autoreactive signature, supporting an association between antigen reactivity, TCR proximity, and molecular profile (Fig. 3B and Fig. S1).

TCR cell lines recognizing the same epitope showed common but not exclusive V and J segments. Four out of five TCRs (80%) that recognized the $SepSecS_{177-204}$ epitope (p23/24) use TRAV8-3 segment, two out of three TCRs (67%) that recognized the $SepSecS_{249-276}$ epitope (p32/33) use TRBV5-4 segment, and two out of three TCRs (67%) that recognized the $SepSecS_{73-100}$ epitope (p10/11) use both TRBV7-2 and TRBJ2-5 segments (Table 2). Also, TRBJ2-5 is shared between six TCRs (35% of TCR cell lines) that react against five distinct epitopes (Table 2). Comparison of TRBV/J gene segment distribution among GLIPH2 groups of interest revealed an enrichment of TRBV7-2, representing 57% of TRBVs in total groups and 72% of TRBVs in TCRs from groups I and II (Fig. 3A, C and Table S7). Moreover, among the 484 SepSecS-specific TCR clonotypes, TRBV7-2 (which encodes for Vβ6.7 chain) was highly represented. It was significantly enriched in patients carrying the *HLA-DPA1*02:01∼HLA-DPB1*01:01* (*DP*2.1*) haplotype, suggesting a potential over-usage of TRBV7-2 by SepSecS-specific autoreactive CD4 T cells during AIH, especially in *DP*2.1-positive* patients (Fig. 3D and Table S3). These results suggest that $SepSecS_{73-100}$, $SepSecS_{177-204}$, and $SepSecS_{249-276}$ epitopes are recognized by multiple autoreactive TCRs with shared CDR3 motifs among AIH patients.

Ex vivo identification of CD4 T cells specific for the $SepSecS_{73-100}$ epitope

Next, we attempted to detect $SepSecS_{73-100}$-reactive CD4 T cells in AIH patients positive for anti-SLA antibodies that carry or not the *HLA-DPA1*02:01∼HLA-DPB1*01:01* (*DP*2.1*) haplotype (Table 1 and Table S8). As previously described,[19,20,30–37] antigen-specific CD4 T cells can be detected after short peptide stimulation *in vitro* through CD154 upregulation. We stimulated PBMCs with a pool of peptides spanning the entire SepSecS sequence (see Patients and methods) or the $SepSecS_{73-100}$ epitope (p10/11) (Fig. 4). We focused on CD154+ PD-1+ subset of CD45RA– memory CD4 T cells (mCD4) which represents the SepSecS-specific CD4 T-cell compartment[19,20] (Fig. 4A, B). All patients had positive SepSecS CD4 T-cell reactivity when PBMCs were stimulated with total SepSecS peptides compared with unstimulated condition, and no association with treatment was found ($median_{none} = 4$, $median_{SepSecS\text{-}total} = 248$, $p = 0.001$; Fig. 4B and Table S8). *DP*2.1-positive* patients (n = 5) seemed to exhibit higher total SepSecS CD4 T-cell reactivity compared with patients with a different haplotype (n = 6); however, this difference was not statistically significant (Fig. 4B–D and Table S8). Compared with unstimulated condition, $SepSecS_{73-100}$ peptide stimulation was associated with significant antigen reactivity in total AIH patients ($median_{none} = 4$, $median_{SepSecS\text{-}73\text{-}100} = 48$, $p = 0.002$; Fig. 4B and Table S8). The CD4 T-cell reactivity against the $SepSecS_{73-100}$ epitope was 10 times higher in *DP*2.1-positive* AIH patients (n = 5) in terms of frequency of CD154+ PD-1+ mCD4 per million mCD4 (Fig. 4C and Table S8). Moreover, $SepSecS_{73-100}$ reactivity represented 15% to 67% of total SepSecS CD4 T-cell reactivity in *DP*2.1-positive* patients, compared with 0% to 29% in other AIH patients (n = 6) (Fig. 4D, E and Table S8). These results suggest that the

reactivity against the $SepSecS_{73-100}$ epitope represents a substantial part of SepSecS reactivity in *DP*2.1-positive* AIH patients.

Discussion

In this study, our goal was to decipher the CD4 T-cell epitope recognition of SepSecS self-antigen and the associated HLA restriction in AIH. Previously, from nine patients, we successfully isolated single cells and determined the TCRβ and/or α sequences of more than 850 CD4 T lymphocytes reacting against SepSecS after brief *in vitro* peptide stimulation.[19,20] Within these datasets, more than 400 TCR clones were identified. To determine dominant CD4 T-cell epitopes, we chose to focus on 17 of the most expanded clones for generating SepSecS-specific TCR cell lines. Using *in vitro* peptide stimulation on reconstructed SepSecS-specific TCR cell lines and class II HLA-blocking antibodies, we identified six reactive amino acid regions, including three ($SepSecS_{73-100}$, $SepSecS_{177-204}$, and $SepSecS_{249-276}$) that were recognized by three or more TCRs with the same HLA restriction. Moreover, we showed that autoreactive TCRs recognized SepSecS epitopes presented not only by HLA-DRB1*03:01 and HLA-DRB1*07:01 molecules, both coded by alleles associated with a higher risk of AIH,[8–10] but also by HLA-DQB1*02:02 molecule and HLA-DPA1*02:01/HLA-DPB1*01:01 complex. Furthermore, TCR clustering analysis using GLIPH2 algorithm suggested that autoreactive TCRs from distinct AIH patients could react against a common epitope based on shared CDR3 motifs, which highlighted the HLA-DPA1*02:01/HLA-DPB1*01:01-restricted $SepSecS_{73-100}$ epitope as a potential highly targeted epitope in our SepSecS-specific TCR dataset. *Ex vivo* peptide stimulation assay showed that this epitope represents a substantial part of total SepSecS CD4 T-cell reactivity in *HLA-DPA1*02:01∼HLA-DPB1*01:01* AIH patients. Although this strategy is time-consuming and technically challenging, it offers a robust and complete analysis of antigen-specific T-cell reactivity.

In this study, the $SepSecS_{209-236}$ sequence (p27/28) includes epitopes that were recognized by three TCRs from three AIH patients with three distinct HLA restrictions (HLA-DRB1*03:01, HLA-DRB1*07:01, and HLA-DQB1*02:02), none with a high epitope binding affinity. This underlines the fact that a single amino acid sequence could contain epitopes presented to distinct TCRs on various HLA molecules. As BLCL 1064 was a *HLA-DRB1*03:01/07:01* heterozygous cell line, *in vitro* validation with *HLA-DRB1*07:01/07:01* homozygous BLCL could confirm the HLA restriction of the $SepSecS_{217-236}$ epitope recognized by clone 1 2020. Furthermore, even though *HLA-DRB1* is the most frequently expressed gene for DRβ chain, we cannot exclude HLA-DRB3, HLA-DRB4, or HLA-DRB5 restrictions for the HLA-DR-restricted SepSecS epitopes identified in this study.

The higher proportion of $SepSecS_{73-100}$-specific reactivity in *DP*2.1* patients supports its HLA restriction defined *in vitro* and *in silico*. Nevertheless, stimulating PBMCs with 20-mer peptides could include responses from cross-reactive T cells or T cells reacting against similar SepSecS epitopes presented by different HLA molecules, as distinct TCRs recognized p10/11 on HLA-DPA1*02:01/HLA-DPB1*01:01 and p11 on HLA-DRB1*03:01. However, only one TCR has been identified as specific for p11 with HLA-DRB1*03:01 restriction in our study. Furthermore, three out of the six *DP*2.1*-negative AIH patients included in *ex vivo* reactivity experiments have an *HLA-DRB1*03:01* allele and showed low $SepSecS_{73-100}$ reactivity both in terms of frequency of cells (0 to 48 CD154+ PD-1+ CD4 T cells per 10^6 mCD4 *vs.* 27 to 691 in *DP*2.1*-positive AIH patients) and proportion of total SepSecS reactivity (0% to 12% of SepSecS reactivity *vs.* 15% to 67% in *DP*2.1*-positive AIH patients). However, it cannot be excluded that p10/11 epitopes could be presented by other HLA molecules, as reactive CD4 T cells were detected in two *HLA-DRB1*03:01*-negative and *HLA-DPB1*01:01*-negative patients (08-002 and 05-001; Fig. 3 and Table S8). Notably, the use of 20-mer overlapping peptides for *ex vivo* identification of CD4 T-cell reactivity against the DP*2.1-restricted $SepSecS_{73-100}$ epitope could have influenced the presented results and HLA-DP-based tetramer technology could confirm the proportion of $SepSecS_{73-100}$-specific response in AIH patients.

Kramer *et al.*[18] demonstrated that multiple blood CD4 T-cell clones reacted against the $SepSecS_{73-100}$ region in nine of the 14 AIH patients studied, without detailing their non-HLA-DR genotypes. Their study and our study collectively suggest that the $SepSecS_{73-100}$ amino acid region could comprise an important proportion of autoreactive CD4 T cells. Although *DP*2.1* has been recently described as a risk haplotype for primary sclerosing cholangitis, another autoimmune liver disease,[38] and 43% (6/14) of AIH patients in the present study carry the *DP*2.1* haplotype, no study till date has provided an overview of *HLA-DP* haplotype in AIH populations. Complete HLA typing of patients could provide important insights into further HLA-restricted epitope discoveries.

Our results revealed over-usage of some TRBVs by SepSecS-specific CD4 T cells during AIH, especially TRBV7-2 (which encodes for Vβ6.7 chain) in *DP*2.1* patients, suggesting preferred TCR chains for liver self-antigen recognition or during autoimmunity. Of note, the over-usage of TRBV7-2 by gluten-specific CD4 T cells in celiac disease, a distinct autoimmune condition, has already been reported.[39,40] Screening of Vβ6.7 chain expression among SepSecS-specific CD4 T cells could confirm this hypothesis and lead to new strategies for enriching a fraction of untouched SepSecS-specific CD4 T cells.

TCR clustering analysis using GLIPH2 algorithm revealed few shared CDR3β motifs across multiple SepSecS-specific TCRs. The two most significant clusters have similar amino acid motifs between positions 3 and 7 (SLQ[TG] and [S]PQTG) of CDR3β. These two clusters included TCRs that recognized the same epitope ($SepSecS_{73-100}$, p10/11), which suggests redundancy between CDR3 region and self-epitope reactivity. However, all TCRs that recognized the same epitope (*e.g.* HLA-DRB1*03:01-restricted $SepSecS_{177-204}$), through *in vitro* epitope discovery or tetramer staining, were not clustered together and exhibited diverse CDR3 motifs. This could be explained by the fact that GLIPH2 algorithm considers only the CDR3 sequence and not V and J segments of α chains, as four of the $SepSecS_{177-204}$-specific TCR cell lines share TRAV8-3 segment. This emphasizes the complexity of self-epitope recognition by autoreactive TCRs and indicates the limit of

sequential-based analysis not considering the conformation of the TCR–MHCp interaction. The probable low affinity of self-antigen for class II HLA can also be a source of error that may disrupt algorithms trained on more conventional interactions between class II HLA molecules, foreign antigens, and TCRs.

Affiliations

[1]*Nantes Université, CHU Nantes, INSERM, Center for Research in Transplantation and Translational Immunology, UMR 1064, Nantes, France;* [2]*Aix Marseille Université, CNRS, INSERM, Centre d'Immunologie de Marseille-Luminy, CIML, Marseille, France;* [3]*Centre d'Investigation Clinique gastro-nutrition, CHU Nantes, Nantes, France;* [4]*INSERM UMR 1307, CNRS UMR 6075, Angers University, CRCI2NA, Nantes, France;* [5]*Service Hepato-gastro-entérologie et Assistance Nutritionnelle, CHU Nantes, Nantes, France;* [6]*Institut des Maladies de l'Appareil Digestif, CHU Nantes, Nantes, France;* [7]*CHU Rennes, Service des maladies du foie, Université Rennes, INSERM, INRAE, Institut NUMECAN, Rennes, France;* [8]*CHRU Tours, Service Hépato-Gastroentérologie, Tours, France;* [9]*CHU Angers, Service Hépato-Gastroentérologie et Oncologie Digestive, Université d'Angers, Laboratoire HIFIH, UPRES EA3859, SFR 4208, Angers, France;* [10]*CHU Poitiers, Service Hépato-Gastroentérologie, Poitiers, France;* [11]*CHD Vendée-La Roche-sur-Yon, Service Hépato-Gastroentérologie, La Roche-sur-Yon, France;* [12]*CHU Brest, Service Hépato-Gastroentérologie, Brest, France;* [13]*AP-HP. Sorbonne Université, Department of Hepatology, Reference Center for Inflammatory Biliary Diseases and Autoimmune Hepatitis CRMR MIVB-H, European Reference Network on Hepatological Diseases, ERN Rare-Liver, Saint-Antoine Hospital, Paris, France;* [14]*Sorbonne Université, INSERM, Institute of Cardiometabolism and Nutrition, ICAN, Centre de Recherche Saint-Antoine, CRSA, Paris, France;* [15]*Fédération Hospitalo-Universitaire Gut, Liver & Microbiome Research, FHU GLIMMER, Paris, France;* [16]*AP-HP, Hôpital Paul-Brousse, Centre Hépato-Biliaire, Unité INSERM 1193, Villejuif, France;* [17]*AP-HP, Hôpital Beaujon, Service d'Hépatologie Adulte, Clichy, France;* [18]*Department of Neurology, CHU Nantes, Nantes, France;* [19]*Center for Translational Immunology, Benaroya Research Institute, Seattle, WA, USA;* [20]*Service Anatomie et Cytologie Pathologiques, CHU Nantes, Nantes, France*

Abbreviations

aa, amino acid; AIH, autoimmune hepatitis; AIM, activation-induced marker assay; BLCL, B-lymphoblastoid cell line; C.ALB, *Candida albicans*; CDR3, complementarity-determining region 3; CTLA4, cytotoxic T-lymphocyte-associated protein 4; CXCL13, chemokine (C-X-C motif) ligand 13; CYP2D6, cytochrome P450 2D6; DP*2.1, HLA-DPA1*02:01/HLA-DPB1*01:01 or *HLA-DPA1*02:01∼HLA-DPB1*01:01*; FTCD, formimidoyltransferase cyclodeaminase; HLA, human leukocyte antigen; IC50, half-maximal inhibitory concentration; LC-1, liver cytosol 1 antibody; LKM-1, liver kidney microsomal type 1 antibody; p#, peptide number; PBMCs, peripheral blood mononuclear cells; PD-1, programmed cell death protein 1 receptor; PDCE2, pyruvate dehydrogenase complex component E2; SLA, soluble liver antigen; SepSecS, O-phosphoserine selenocysteine tRNA synthase; TCR, T-cell receptor; TIGIT, T-cell immunoreceptor with Ig and ITIM domains; UMAP, uniform manifold approximation and projection.

Financial support

This work was supported by the Agence Nationale de la Recherche (ANR-19 CE17-0024, ANR-24 CE15-7123, ANR-24 CE17-7666), by institutional grants from Institut national de la santé et de la recherche médicale (INSERM) and by the patient association pour la Lutte contre les maladies inflammatoires du foie et des voies biliaires (ALBI), the Fondation Maladies Rares, the Région pays de la Loire, the LabEx IGO program (n° ANR-11-LABX-0016) funded by the Investment into the Future French Government program managed by the Agence Nationale de la Recherche (ANR).

Conflicts of interest

Sara Lemoinne reports consulting work for Mirum, Ipsen, Amgen and Servier, lectures for Ipsen, Mirum, Echosens and congress participation by invitation from Mirum, Ipsen, AbbVie, Gilead. The authors declare no conflicts of interest related to this work.

Please refer to the accompanying ICMJE disclosure forms for further details.

Authors' contributions

TG: performed the experiments, analyzed the data, and wrote the manuscript; AG, LB, VH, LG, SA, and JPJ: performed the experiments; CD and LD: analyzed the data; PJG: performed the experiment and analyzed the data; CC: managed human AIH sample collection and patient database; BC: provided Epstein-Barr virus B-lymphoblastoid cell lines; MK, EBJ, LE, AL, CS, MS, FT, SL, EDM, and AP: provided human AIH samples and critical insight in AIH pathology; FV: managed human AIH sample collection; DAL: provided critical insight in the study design; WWK: provided anti-HLA-DQ-blocking antibody and critical insight in TCR analysis; SB: provided critical insight in the study design; JFM: provided critical insight in AIH pathology; JG: provided human AIH samples, critical insight in AIH pathology, and direction in the study design; PM and SC: designed the study, supervised data analysis, and wrote the manuscript; AR: designed the study, supervised data analysis, performed the experiments, analyzed the data, and wrote the manuscript. All authors reviewed and approved the manuscript.

Data availability

Raw numbers for charts and graphs, and FCS data from human patients' samples are available on demand.

Acknowledgements

We thank he Agence Nationale de la Recherche (ANR), Institut national de la santé et de la recherche médicale (INSERM), the patient association pour la Lutte contre les maladies inflammatoires du foie et des voies biliaires (ALBI), the Fondation Maladies Rares, the Région pays de la Loire, the LabEx IGO program for financial support. We thank the biological resource center for biobanking (CHU Nantes, Hôtel Dieu, Center de ressources biologiques [CRB], Nantes, F-44093, France [BRIF: BB-0033-00040]). We thank all the members of the HEP-ATIMGO network. We thank Dr. Sarah Habes, Dr. Maëva Salimon, and Dr. Annie Lim for the inclusion of new AIH patients. We thank Dr. K. Dornmair for providing the $58\alpha^{-}\beta^{-}$ T-cell hybridoma. We acknowledge Dr. Nicolas Vince for his HLA expertise. We thank the Laboratoire HLA EFS Centre Pays de la Loire for HLA typing of patients.

Supplementary data

Supplementary data to this article can be found online at https://doi.org/10.1016/j.jhepr.2026.101952.

References

Author names in bold designate shared co-first authorship

[1] European Association for the Study of the Liver. EASL Clinical Practice Guidelines on the management of autoimmune hepatitis. J Hepatol 2025;83:453–501.

[2] van Gerven NMF, Verwer BJ, Witte BI, et al. Relapse is almost universal after withdrawal of immunosuppressive medication in patients with autoimmune hepatitis in remission. J Hepatol 2013;58:141–147.

[3] Hartl J, Ehlken H, Weiler-Normann C, et al. Patient selection based on treatment duration and liver biochemistry increases success rates after treatment withdrawal in autoimmune hepatitis. J Hepatol 2015;62:642–646.

[4] Terziroli Beretta-Piccoli B, Mieli-Vergani G, Vergani D. Autoimmmune hepatitis. Cell Mol Immunol 2022;19:158–176.

[5] Kirstein MM, Metzler F, Geiger E, et al. Prediction of short- and long-term outcome in patients with autoimmune hepatitis. Hepatology 2015;62:1524–1535.

[6] Zachou K, Weiler-Normann C, Muratori L, et al. Permanent immunosuppression in SLA/LP-positive autoimmune hepatitis is required although overall response and survival are similar. Liver Int 2020;40:368–376.

[7] Ma Y, Okamoto M, Thomas MG, et al. Antibodies to conformational epitopes of soluble liver antigen define a severe form of autoimmune liver disease. Hepatology 2002;35:658–664.

[8] Donaldson PT, Doherty DG, Hayllar KM, et al. Susceptibility to autoimmune chronic active hepatitis: human leukocyte antigens DR4 and A1-B8-DR3 are independent risk factors. Hepatology 1991;13:701–706.

[9] **Ma Y**, **Su H**, Yuksel M, et al. Human leukocyte antigen profile predicts severity of autoimmune liver disease in children of European ancestry. Hepatology 2021;74:2032–2046.
[10] Baharlou R, Faghihi-Kashani A, Faraji F, et al. HLA-DRB1 alleles of susceptibility and protection in Iranians with autoimmune hepatitis. Hum Immunol 2016;77:330–335.
[11] Cardon A, Conchon S, Renand A. Mechanisms of autoimmune hepatitis. Curr Opin Gastroenterol 2021;37:79–85.
[12] Toda G, Zeniya M, Watanabe F, et al. Present status of autoimmune hepatitis in Japan–correlating the characteristics with international criteria in an area with a high rate of HCV infection. Japanese National Study Group of Autoimmune Hepatitis. J Hepatol 1997;26:1207–1212.
[13] Lim YS, Oh HB, Choi SE, et al. Susceptibility to type 1 autoimmune hepatitis is associated with shared amino acid sequences at positions 70-74 of the HLA-DRB1 molecule. J Hepatol 2008;48:133–139.
[14] Pando M, Larriba J, Fernandez GC, et al. Pediatric and adult forms of type I autoimmune hepatitis in Argentina: evidence for differential genetic predisposition. Hepatology 1999;30:1374–1380.
[15] Umemura T, Katsuyama Y, Yoshizawa K, et al. Human leukocyte antigen class II haplotypes affect clinical characteristics and progression of type 1 autoimmune hepatitis in Japan. PLoS One 2014;9:e100565.
[16] **Mix H**, **Weiler-Normann C**, Thimme R, et al. Identification of CD4 T-cell epitopes in soluble liver antigen/liver pancreas autoantigen in autoimmune hepatitis. Gastroenterology 2008;135:2107–2118.
[17] **Zhao Y**, **Zhang Y**, Liu Y, et al. Identification of T cell epitopes on soluble liver antigen in Chinese patients with auto-immune hepatitis. Liver Int 2011;31:721–729.
[18] Kramer M, Mele F, Jovic S, et al. Clonal analysis of SepSecS-specific B and T cells in autoimmune hepatitis. J Clin Invest 2025;135:e183776.
[19] Renand A, Cervera-Marzal I, Gil L, et al. Integrative molecular profiling of autoreactive CD4 T cells in autoimmune hepatitis. J Hepatol 2020;73:1379–1390.
[20] **Cardon A**, **Guinebretière T**, Dong C, et al. Single cell profiling of circulating autoreactive CD4 T cells from patients with autoimmune liver diseases suggests tissue imprinting. Nat Commun 2025;16:1161.
[21] European Association for the Study of the Liver. EASL clinical practice guidelines: autoimmune hepatitis. J Hepatol 2015;63:971–1004.
[22] Hennes EM, Zeniya M, Czaja AJ, et al. Simplified criteria for the diagnosis of autoimmune hepatitis. Hepatology 2008;48:169–176.
[23] Letourneur F, Malissen B. Derivation of a T cell hybridoma variant deprived of functional T cell receptor alpha and beta chain transcripts reveals a nonfunctional alpha-mRNA of BW5147 origin. Eur J Immunol 1989;19:2269–2274.
[24] Blank U, Boitel B, Mège D, et al. Analysis of tetanus toxin peptide/DR recognition by human T cell receptors reconstituted into a murine T cell hybridoma. Eur J Immunol 1993;23:3057–3065.
[25] Seitz S, Schneider CK, Malotka J, et al. Reconstitution of paired T cell receptor alpha- and beta-chains from microdissected single cells of human inflammatory tissues. Proc Natl Acad Sci U S A 2006;103:12057–12062.
[26] **Kaabinejadian S**, **Barra C**, Alvarez B, et al. Accurate MHC motif deconvolution of immunopeptidomics data reveals a significant contribution of DRB3, 4 and 5 to the total DR immunopeptidome. Front Immunol 2022;13:835454.
[27] **Glanville J**, **Huang H**, Nau A, et al. Identifying specificity groups in the T cell receptor repertoire. Nature 2017;547:94–98.
[28] **Huang H**, **Wang C**, Rubelt F, et al. Analyzing the Mycobacterium tuberculosis immune response by T-cell receptor clustering with GLIPH2 and genome-wide antigen screening. Nat Biotechnol 2020;38:1194–1202.
[29] Mayer-Blackwell K, Schattgen S, Cohen-Lavi L, et al. TCR meta-clonotypes for biomarker discovery with tcrdist3 enabled identification of public, HLA-restricted clusters of SARS-CoV-2 TCRs. Elife 2021;10:e68605.
[30] **Saggau C**, **Bacher P**, Esser D, et al. Autoantigen-specific CD4+ T cells acquire an exhausted phenotype and persist in human antigen-specific autoimmune diseases. Immunity 2024;57:2416. 32.e8.
[31] Renand A, Archila LD, McGinty J, et al. Chronic cat allergen exposure induces a TH2 cell-dependent IgG4 response related to low sensitization. J Allergy Clin Immunol 2015;136:1627. 35.e13.
[32] Renand A, Farrington M, Whalen E, et al. Heterogeneity of Ara h Component-Specific CD4 T cell responses in peanut-allergic subjects. Front Immunol 2018;9:1408.
[33] Renand A, Newbrough S, Wambre E, et al. Arginine kinase Pen m 2 as an important shrimp allergen recognized by TH2 cells. J Allergy Clin Immunol 2014;134:1456. 9.e7.
[34] **Renand A**, **Shamji MH**, Harris KM, et al. Synchronous immune alterations mirror clinical response during allergen immunotherapy. J Allergy Clin Immunol 2018;141:1750–17560.e1.
[35] Archila LD, Chow I-T, McGinty JW, et al. Ana o 1 and Ana o 2 cashew allergens share cross-reactive CD4(+) T cell epitopes with other tree nuts. Clin Exp Allergy 2016;46:871–883.
[36] Bacher P, Schink C, Teutschbein J, et al. Antigen-reactive T cell enrichment for direct, high-resolution analysis of the human naive and memory Th cell repertoire. J Immunol 2013;190:3967–3976.
[37] Chattopadhyay PK, Yu J, Roederer M. A live-cell assay to detect antigen-specific CD4+ T cells with diverse cytokine profiles. Nat Med 2005;11:1113–1117.
[38] Zecher BF, Ellinghaus D, Schloer S, et al. HLA-DPA1*02:01~B1*01:01 is a risk haplotype for primary sclerosing cholangitis mediating activation of NKp44+ NK cells. Gut 2024;73:325–337.
[39] Qiao SW, Ráki M, Gunnarsen KS, et al. Posttranslational modification of gluten shapes TCR usage in celiac disease. J Immunol 2011;187:3064–3071.
[40] Qiao SW, Christophersen A, Lundin KEA, et al. Biased usage and preferred pairing of α- and β-chains of TCRs specific for an immunodominant gluten epitope in coeliac disease. Int Immunol 2014;26:13–19.

Keywords: Autoimmune hepatitis; Liver immunology; SepSecS; Epitope; Autoreactive CD4 T cells; HLA restriction; GLIPH2; HLA-DPA1*02:01/HLA-DPB1*01:01.
Received 22 September 2025; received in revised form 25 June 2026; accepted 26 June 2026; Available online xxx

Journal of Hepatology, Volume *8*

Supplemental information

Mapping of class II HLA restriction of SepSecS-specific CD4 T-cell epitopes in autoimmune hepatitis

Thomas Guinebretière, Alexandra Garcia, Chuang Dong, Laura Bernier, Virginie Huchet, Pierre-Jean Gavlovsky, Laurine Gil, Sakina Ado, Léa David, Caroline Chevalier, Jean-Paul Judor, Béatrice Clémenceau, Marion Khaldi, Edouard Bardou-Jacquet, Laure Elkrief, Adrien Lannes, Christine Silvain, Matthieu Schnee, Florence Tanne, Sara Lemoinne, Eleonora De Martin, Aurélie Plessier, Fabienne Vavasseur, David-Axel Laplaud, William W. Kwok, Sophie Brouard, Jean-François Mosnier, Jérôme Gournay, Pierre Milpied, Sophie Conchon, and Amédée Renand

Mapping of class II HLA restriction of SepSecS-specific CD4 T cell epitopes in autoimmune hepatitis

Thomas Guinebretière, Alexandra Garcia, Chuang Dong, Laura Bernier, Virginie Huchet, Pierre-Jean Gavlovsky, Laurine Gil, Sakina Ado, Léa David, Caroline Chevalier, Jean-Paul Judor, Béatrice Clémenceau, Marion Khaldi, Edouard Bardou-Jacquet, Laure Elkrief, Adrien Lannes, Christine Silvain, Matthieu Schnee, Florence Tanne, Sara Lemoinne, Eleonora De Martin, Aurélie Plessier, Fabienne Vavasseur, David-Axel Laplaud, William W. Kwok, Sophie Brouard, Jean-François Mosnier, Jérôme Gournay, Pierre Milpied, Sophie Conchon, Amédée Renand

Fig. S1...2
Table S1...3
Table S2...4
Table S3...separate excel file
Table S4...4
Table S5...separate excel file
Table S6...5
Table S7...separate excel file
Table S8...6
Supplementary references...7

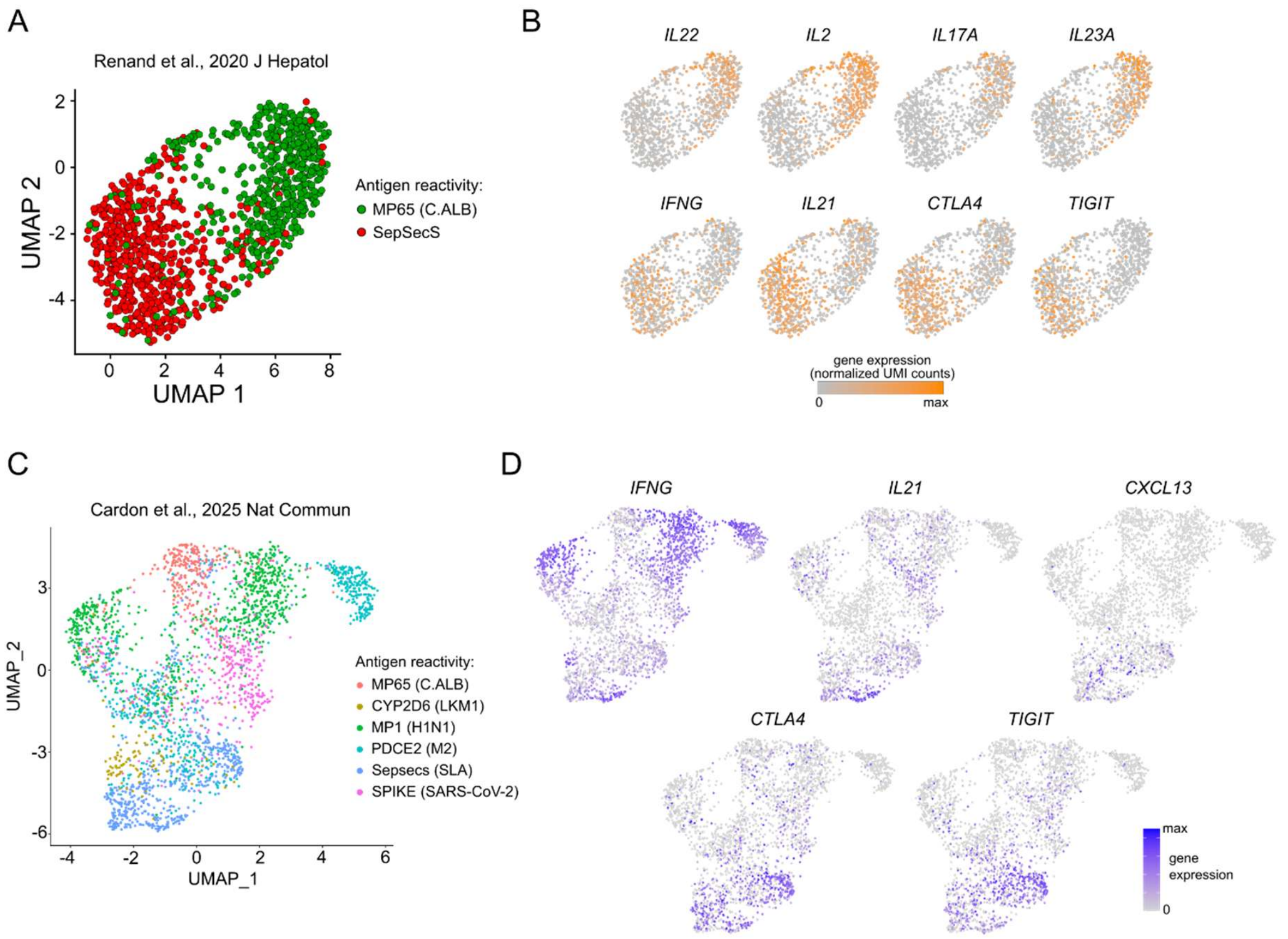

Fig. S1. Molecular profile of antigen-specific CD4 T cells from Renand et al., 2020 J Hepatol and Cardon et al., 2025 Nat Commun. (A) UMAP representation of single-cell transcriptomes of antigen-specific CD4 T cells from Renand et al., 2020 J Hepatol, colored by antigen reactivity (MP65-specific CD4 T cells in green, SepSecS-specific CD4 T cells in red). (B) UMAP representation used in A colored by expression of indicated genes. (C) UMAP representation of single-cell transcriptomes of antigen-specific CD4 T cells from Cardon et al., 2025 Nat Commun, colored by antigen reactivity as indicated. (D) UMAP representation used in C colored by expression of indicated genes.

Table S1. Amino acid sequence of SepSecS peptides.

peptide sequence (red=false, blue=substitution)	peptide number (p#)	position on AAD33963 (used in the manuscript)	position on Q9HD40 (curated sequence)	pool number	reference if published
MSTSYGCFWRRFIHGIGRSG	p1	1-20	/	1	
WRRFIHGIGRSGDISAVQPK	p2	9-28	90-107	1	
GRSGDISAVQPKAAGSSLLN	p3	17-36	96-115	1	
VQPKAAGSSLLNKITNSLVL	p4	25-44	104-123	1	
SLLNKITNSLVLDIIKLAGV	p5	33-52	112-131	1	
SLVLDIIKLAGVHTVANCFV	p6	41-60	120-139	2	[1]
LAGVHTVANCFVVPMATGMS	p7	49-68	128-147	2	
NCFVVPMATGMSLTLCFLTL	p8	57-76	136-155	2	[1]
TGMSLTLCFLTLRHKRPKAK	p9	65-84	144-163	2	[1]
FLTLRHKRPKAKYIIWPRID	p10	73-92	152-171	2	[2,3]
PKAKYIIWPRIDQKSCFKSM	p11	81-100	160-179	3	[2,3]
PRIDQKSCFKSMITAGFEPV	p12	89-108	168-187	3	[2]
FKSMITAGFEPVVIENVLEG	p13	97-116	176-195	3	[2]
FEPVVIENVLEGDELRTDLK	p14	105-124	184-203	3	[2]
VLEGDELRTDLKAVEAKVQE	p15	113-144	192-211	3	
TDLKAVEAKVQELGPDCILC	p16	121-140	200-219	4	
KVQELGPDCILCIHSTTSCF	p17	129-148	208-227	4	
CILCIHSTTSCFAPRVPDRL	p18	137-156	216-235	4	
TSCFAPRVPDRLEELAVICA	p19	145-164	224-243	4	
PDRLEELAVICANYDIPHIV	p20	153-173	232-251	4	
VICANYDIPHIVNNAYGVQS	p21	161-180	240-259	5	
PHIVNNAYGVQSSKCMHLIQ	p22	169-188	248-267	5	
GVQSSKCMHLIQQGARVGRI	p23	177-196	256-275	5	[2–5]
HLIQQGARVGRIDAFVQSLD	p24	185-204	264-283	5	[2–5]
VGRIDAFVQSLDKNFMVPVG	p25	193-212	272-291	5	
QSLDKNFMVPVGGAIIAGFN	p26	201-220	280-299	6	
VPVGGAIIAGFNDSFIQEIS	p27	209-228	288-307	6	
AGFNDSFIQEISKMYPGRAS	p28	217-236	296-315	6	
QEISKMYPGRASASPSLDVL	p29	225-244	304-323	6	
GRASASPSLDVLITLLSLGS	p30	233-252	312-331	6	
LDVLITLLSLGSNGYKKLLK	p31	241-260	320-339	7	
SLGSNGYKKLLKERKEMFSY	p32	249-268	328-347	7	
KLLKERKEMFSYLSNQIKKL	p33	257-276	336-355	7	[4]
MFSYLSNQIKKLSEAYNERL	p34	265-284	344-363	7	
IKKLSEAYNERLLHTPHNPI	p35	273-292	352-371	7	
NERLLHTPHNPISLAMTLKT	p36	281-300	360-379	8	
HNPISLAMTLKTLDEHRDKA	p37	289-308	368-387	8	
TLKTLDEHRDKAVTQLGSML	p38	297-316	376-395	8	
RDKAVTQLGSMLFTKQVSGA	p39	305-324	384-403	8	[1]
GSMLFTKQVSGARVVPLGSM	p40	313-332	392-411	8	
VSGARVVPLGSMQTVSGYTF	p41	321-340	400-419	9	[1]
LGSMQTVSGYTFRGFMSHTN	p42	329-348	408-427	9	
GYTFRGFMSHTNNYPCAYLN	p43	337-356	416-435	9	
SHTNNYPCAYLNAASAIGMK	p44	345-364	424-443	9	
AYLNAASAIGMKMQDVDLFI	p45	353-372	432-451	9	
IGMKMQDVDLFINRLDRCLK	p46	361-380	440-459	10	
DLFINRLDRCLKAVRKERSK	p47	369-388	448-467	10	[4]
RCLKAVRKERSKESDDNYDK	p48	377-396	456-475	10	
ERSKESDDNYDKTEDVDIEE	p49	385-404	464-483	10	
NYDKTEDVDIEEMALKLDNV	p50	393-412	472-491	10	
DIEEMALKLDNVLLDTYQDA	p51	401-420	480-499	11	
LDNVLLDTYQDASS	p52	409-422	488-501	11	
EEMALKLDNVLLDTYQDASS	p53	403-422	482-501	11	

42

Table S2. List of antibodies.

antibody name	clone	antigen	brand	reference	dilution
Alexa Fluor 488 anti-human CD4	OKT4	CD4	BioLegend	317420	20
PE anti-human CD154	5C8	CD154	Miltenyi	130-113-607	20
PerCP/Cyanine5.5 anti-human CD279 (PD-1)	EH12.2H7	CD279	BioLegend	329914	20
APC/Cyanine7 anti-human CD45RA	HI100	CD45RA	BioLegend	304127	20
Brilliant Violet 510 anti-human CD3	OKT3	CD3	BioLegend	317332	20

Table S3. List of TCR clonotypes from scRNA-seq data.

See Table S3.xlsx.

Table S4. Class II HLA genotypes of Epstein-Barr Virus B lymphoblastoid cell lines (BLCL).

BLCL ID	HLA-DRB1	HLADQB1	HLADPA1	HLADPB1
BLCL FEB*DR3	03:01 / 03:01	02:01 / 02:01	NA	04:01 / 04:01
BLCL 1064	03:01 / 07:01	02:01 / 02:02	01:03 / 02:01	01:01 / 04:01

NA: no data available.

Table S5. Epitope binding predictions using NetMHCIIpan 4.1 BA tool.

See Table S5.xlsx.

Table S6. GLIPH2 clustering of TCRs from SepSecS$_{187\text{-}197}$-loaded HLA-DRB1*03:01 tetramer-specific CD4 T cells and clones recognizing SepSecS$_{177\text{-}204}$ epitope *in vitro.*

Group	TCR cell line	Epitope	index	Pattern	CDR3β	Vβ	Jβ	CDR3α	Patient ID
I			$1_{2,3,4,5}$	(Q)GVYG	CASSESSQGVYGYTF	TRBV6-1	TRBJ1-2	NA	01-004
			$1_{4,5}$	GVYG	CATSSQTGGVYGYTF	TRBV15	TRBJ1-2	CAVDRGSGYSTLTF	01-004
			$1_{4,5}$	GVYG	CATSSQTGGVYGYTF	TRBV15	TRBJ1-2	CAVGSGGYNKLIF	01-004
			$1_{4,5}$	GVYG	CATSSQTGGVYGYTF	TRBV15	TRBJ1-2	NA	01-004
			$1_{2,3,4,5}$	(Q)GVYG	CASSSTRQGVYGYTF	TRBV7-9	TRBJ1-2	CAFTNFGNEKLTF	01-004
			$1_{2,3,4,5}$	(Q)GVYG	CASSSTRQGVYGYTF	TRBV7-9	TRBJ1-2	NA	01-128
			$1_{2,3,4,5}$	(Q)GVYG	CASSSTRQGVYGYTF	TRBV7-9	TRBJ1-2	CAVGDAGYSTLTF	01-004
	clone 6 2020	SepSecS$_{177\text{-}204}$ (p23/24)	$1_{2,3,4,5}$	(Q)GVYG	CASSFPGQGVYGYTF	TRBV7-2	TRBJ1-2	CAVGSGGYNKLIF	01-018
			3	QGV	CATSSPGQGVQPQHF	TRBV15	TRBJ1-5	CAVGPGGYNKLIF	01-018
II	clone 7 2020	SepSecS$_{177\text{-}204}$ (p23/24)	8	single	CASSSFDRGNYGYTF	TRBV7-3	TRBJ1-2	CAVTPSGGYQKVTF	01-018
			8	single	CASSSFDRGNYGYTF	TRBV7-3	TRBJ1-2	CAGGPNKLIF	01-018
III			41	single	CASSRRLVSSYNEQFF	TRBV5-4	TRBJ2-1	CAASMVDTGRRALTF	01-128
			41	single	CASSRRLVSSYNEQFF	TRBV5-4	TRBJ2-1	CAVSPNFGNEKLTF	01-128
			41	single	CASSRRLVSSYNEQFF	TRBV5-4	TRBJ2-1	NA	01-128
IV			52	single	CSARYPLRLGNTIYF	TRBV20-1	TRBJ1-3	CAVDGANDYKLSF	01-046
			52	single	CSARYPLRLGNTIYF	TRBV20-1	TRBJ1-3	NA	01-004

index: X_Y where X is the principal index (red pattern) and Y is the additional index(es) (blue pattern); single: exact same CDR3β sequence; NA: no data available.

Table S7. Output data from GLIPH2 TCR clustering analysis shown in Fig. 3A.

See Table S7.xlsx.

Table S8. Clinical characteristics and SepSecS reactivity of patients used in Fig. 4.

Patient ID	*DP*2.1*	Age	Sex	AST (UI/L)	ALT (UI/L)	IgG (g/L)	Treatment	Nb CD154+ PD-1+ mCD4 per 10^6 mCD4		
								$SepSecS_{total}$	$SepSecS_{73-100}$	none
01-051	no	36	F	47	52	17,3	Azathioprin	1166	48	12
05-004	no	45	F	21	17	11,8	Steroids / MMF	223	0	0
07-009	no	54	F	58	58	45	Azathioprin	248	18	6
08-002	no	45	F	20	20	ND	Azathioprin	366	84	0
01-354	no	59	F	115	167	18	No treatment	97	11	0
05-001	no	58	F	38	32	16,8	MMF	163	47	1
01-046	yes	65	F	21	13	10,5	Azathioprin	179	27	10
01-165	yes	34	F	53	104	16,6	No treatment	1478	586	34
01-018	yes	38	F	60	64	20,2	Azathioprin	1200	691	7
01-004	yes	39	F	21	16	13,4	Steroids / Azathioprin	2348	349	4
01-128	yes	33	F	21	18	13,1	Azathioprin	145	98	0

*DP*2.1*: *HLA-DPA1*02:01~HLA-DPB1*01:01*; AST: aspartate transaminase; ALT: alanine transaminase; IgG: type G immunoglobulin; Nb: number; mCD4: CD45RA- CD4+ CD3+ T cells; F: female; MMF: mycophenolate mofetil; ND: not determined.

Supplementary references

Author names in bold designate shared co-first authorship

[1] **Zhao Y**, **Zhang Y**, Liu Y, et al. Identification of T cell epitopes on soluble liver antigen in Chinese patients with auto-immune hepatitis. Liver Int 2011;31:721–9.

[2] Kramer M, Mele F, Jovic S, et al. Clonal analysis of SepSecS-specific B and T cells in autoimmune hepatitis. J Clin Invest 2025;135:e183776.

[3] Renand A, Cervera-Marzal I, Gil L, et al. Integrative molecular profiling of autoreactive CD4 T cells in autoimmune hepatitis. J Hepatol 2020;73:1379–90.

[4] **Mix H**, **Weiler–Normann C**, Thimme R, et al. Identification of CD4 T-Cell Epitopes in Soluble Liver Antigen/Liver Pancreas Autoantigen in Autoimmune Hepatitis. Gastroenterology 2008;135:2107–18.

[5] **Cardon A**, **Guinebretière T**, Dong C, et al. Single cell profiling of circulating autoreactive CD4 T cells from patients with autoimmune liver diseases suggests tissue imprinting. Nat Commun 2025;16:1161.
