# Supplementary material for: Mapping of class II HLA restriction of SepSecS-specific CD4 T-cell epitopes in autoimmune hepatitis
Source: JHEP Rep. 2026 Aug 11;8(9):101952. doi: 10.1016/j.jhepr.2026.101952 (PMC13482648; doi:10.1016/j.jhepr.2026.101952)
Supplement: Supplementary file 4 [file mmc2.docx]

**JHEP Reports**

**CTAT methods**

Tables for a “Complete, Transparent, Accurate and Timely account” (CTAT) are now mandatory for all revised submissions. The aim is to enhance the reproducibility of methods.

- Only include the parts relevant to your study
- Refer to the CTAT in the main text as ‘Supplementary CTAT Table’
- Do not add subheadings
- Add as many rows as needed to include all information
- Only include one item per row

**If the CTAT form is not relevant to your study, please outline the reasons why:**

1.1 **Antibodies**

| **Name** | **Citation** | **Supplier** | **Cat no.** | **Clone no.** |
| --- | --- | --- | --- | --- |
| **Alexa Fluor 488 anti-human CD4** |  | **BioLegend** | **317420** | **OKT4** |
| **PE anti-human CD154** |  | **Miltenyi** | **130-113-607** | **5C8** |
| **PerCP/Cyanine5.5 anti-human CD279 (PD-1)** |  | **BioLegend** | **329914** | **EH12.2H7** |
| **APC/Cyanine7 anti-human CD45RA** |  | **BioLegend** | **304127** | **HI100** |
| **Brilliant Violet 510 anti-human CD3** |  | **BioLegend** | **317332** | **OKT3** |
| **anti-CD40** |  | **Miltenyi** | **130-094-133** | **HB14** |
| **anti-PE magnetic beads** |  | **Miltenyi** | **130-048-801** |  |
| **anti-HLA-DR blocking antibody** |  | **BD Pharmingen** | **555809** | **G46-6** |
| **anti-HLA-DQ blocking antibody** |  | **gift from Dr. W. W. Kwok** |  | **SPVL3** |
| **anti-HLA-DP blocking antibody** |  | **Leinco Technologies** | **H266-200UG** | **B7/21** |

1.2 **Cell lines**

| **Name** | **Citation** | **Supplier** | **Cat no.** | **Passage no.** | **Authentication test method** |
| --- | --- | --- | --- | --- | --- |
| **T cell hybridoma 58α-β-** | **Blank, Uet al., Eur. J. Immunol. 23, 3057–3065 (1993)** |  |  |  | **gift from Pr Klaus Dornmair** |

1.3 **Organisms**

| **Name** | **Citation** | **Supplier** | **Strain** | **Sex** | **Age** | **Overall n number** |
| --- | --- | --- | --- | --- | --- | --- |
|  |  |  |  |  |  |  |

1.4 **Sequence based reagents**

| **Name** | **Sequence** | **Supplier** |
| --- | --- | --- |
| **SepSecS** | **AAD33963** | **Synpeptide** |

1.5 **Biological samples**

| **Description** | **Source** | **Identifier** |
| --- | --- | --- |
| **PBMCs from autoimmune liver disease patients** | **Bio Bank CHU of Nantes** | **BIO-MAI-FOIE** |

1.6 **Deposited data**

| **Name of repository** | **Identifier** | **Link** |
| --- | --- | --- |
| **GSE149298** | **Integrative molecular profiling of autoreactive CD4 T cells in autoimmune hepatitis** | **https://www.ncbi.nlm.nih.gov/geo/query/acc.cgi** |
| **GSE270739** | **Single-cell analysis of circulating autoreactive CD4 T cells of autoimmune hepatitis patients** | **https://www.ncbi.nlm.nih.gov/geo/query/acc.cgi** |

1.7 **Software**

| **Software name** | **Manufacturer** | **Version** |
| --- | --- | --- |
| **NetMHCIIpan** | **IEDB analysis resource** | **4.1** |
| **GLIPH2** |  | **2.0** |
| **GraphPad Prism software** | **GraphPad Software** | **6.0** |
| **FlowJo** | **FlowJo** | **10.7.1** |
| **Omiq** | **Dotmatics** |  |

1.8 **Other (*e.g*. drugs, proteins, vectors etc.)**

| **pRc/RSV expression vector** | **Life technologies** |  |
| --- | --- | --- |
|  |  |  |

1.9 **Please provide the details of the corresponding methods author for the manuscript:**

**Amedee Renand: amedee.renand@univ-nantes.fr**

**Thomas Guinebretière: thomasguinebretiere@univ-nantes.fr**

**2.0	Please confirm for randomised controlled trials all versions of the clinical protocol are included in the submission. These will be published online as supplementary information.**
